# Mitigating Goodhart’s law in epitope-conditioned TCR generation using plug-and-play reward designs

**DOI:** 10.1093/bioinformatics/btag499

**Published:** 2026-08-21

**Authors:** Pengfei Zhang, Xiaoyi He, Fredo Guan, Hao Mei, Gloria Grama, Seojin Bang, Heewook Lee

**Affiliations:** School of Computing Augmented Intelligence, Arizona State University, Tempe, AZ 85281, United States; Biodesign Institute, Arizona State University, Tempe, AZ 85281, United States; School of Computing Augmented Intelligence, Arizona State University, Tempe, AZ 85281, United States; Biodesign Institute, Arizona State University, Tempe, AZ 85281, United States; School of Computing Augmented Intelligence, Arizona State University, Tempe, AZ 85281, United States; Biodesign Institute, Arizona State University, Tempe, AZ 85281, United States; School of Computing Augmented Intelligence, Arizona State University, Tempe, AZ 85281, United States; Biodesign Institute, Arizona State University, Tempe, AZ 85281, United States; Biodesign Institute, Arizona State University, Tempe, AZ 85281, United States; Elorian AI, CA 94301, United States; School of Computing Augmented Intelligence, Arizona State University, Tempe, AZ 85281, United States; Biodesign Institute, Arizona State University, Tempe, AZ 85281, United States

## Abstract

**Motivation:**

Epitope-conditioned T cell receptor (TCR) generation extends protein language modeling to the design of therapeutically relevant receptors. Reinforcement learning (RL) post-training with surrogate binding predictors can improve generation controllability, but it is vulnerable to Goodhart’s Law: optimizing an imperfect surrogate reward can lead to reward inflation, distributional drift, and biologically implausible or nonspecific sequences. We investigate whether reward hacking can be mitigated through improved reward formulations without modifying the generator architecture or training pipeline and without requiring additional training data.

**Results:**

We introduce a plug-and-play reward-design framework for RL-based TCR generation that combines heuristic biological priors, model ensembling, and binding-specificity objectives based on max-margin and contrastive formulations. These components suppress degenerate sequences, reduce model-specific biases, and discourage cross-epitope binding. During RL fine-tuning, the proposed rewards stabilize optimization, limit surrogate-reward inflation, preserve canonical CDR3β sequence patterns and repertoire diversity, and maintain closer alignment with experimentally validated TCR-binder distributions. In evaluations on unseen epitopes, the specificity-aware reward formulations provide the strongest overall performance, improving diversity and ground-truth distributional alignment while retaining biological authenticity and predicted target binding. These findings demonstrate that Goodhart-resistant reward design improves the reliability, controllability, and generalization of epitope-conditioned TCR generation.

**Availability and implementation:**

Code and models are available in a public repository (https://github.com/Lee-CBG/TCRRobustRewardDesign).

## 1. Introduction

T cells play a central role in adaptive immunity by recognizing and eliminating abnormal or infected cells. The recognition is mediated by T cell receptors (TCRs) that bind to short peptide fragments (epitopes) presented by the major histocompatibility complex (MHC) on target cells Davis and Bjorkman (1988); Rudolph *et al.* (2006). The design of epitope-specific TCRs holds great promise for personalized cancer immunotherapy and the treatment of other infectious diseases, yet experimental discovery and screening remain prohibitively slow, costly, and limited in throughput Chandran and Klebanoff (2019).

Recent advances in protein language modeling have shifted computational immunology beyond the prediction of epitope–TCR binding affinity toward the more ambitious task of epitope-conditioned TCR generation, where the goal is to generate candidate TCRs capable of binding to a target epitope. Most existing methods treat this as a prompt–completion task, using an epitope as context and generating TCRs auto-regressively. These models are typically trained via supervised fine-tuning on known epitope–TCR pairs using a next-token prediction objective. Since the next-token objective maximizes only sequence likelihood, it does not guarantee key biological properties such as epitope specificity, repertoire plausibility, or structural feasibility. As a result, the model reproduces dataset biases rather than meaningful biological patterns, producing sequences that lack interpretability and offer limited value for practical TCR design.

To improve controllability, recent TCR generation methods have adapted reinforcement learning from human feedback (RLHF) frameworks, originally developed for large language models Christiano *et al.* (2017); Ziegler *et al.* (2019); Ouyang *et al.* (2022), to the problem of biological sequence design Angermueller *et al.* (2019); Yang *et al.* (2019). In this paradigm, a pretrained auto-regressive generator acts as a policy that produces candidate sequences, a reward model evaluates these sequences based on desired properties, and the policy is updated to increase the likelihood of high-reward samples.

Due to the high cost of obtaining human- or expert-validated labels, RLHF applied to many domains, including LLMs, relies on surrogate reward models trained on limited human preferences Ouyang *et al.* (2022). The challenge is far more severe in the biological domain. In NLP, large-scale preference datasets enable reward models that approximate human judgment reasonably well, whereas in biology, the true “golden reward” signal of experimental validation of molecular fitness or binding efficacy is scarce and costly to obtain. As a result, RLHF-style TCR generation must also rely on surrogate reward models, typically on binding affinity prediction (BAP) networks trained on limited TCR–epitope interaction data, which provide imperfect, and potentially misleading approximations of true biological fitness.

The misalignment between a surrogate reward and the true biological objective introduces a well-known failure mode captured by Goodhart’s Law Goodhart (1984); Manheim and Garrabrant (2018): *“When a measure becomes a target, it ceases to be a good measure.”* When an imperfect surrogate model is optimized as a reward, it becomes susceptible to reward hacking Skalse *et al.* (2022), where the generator exploits shortcuts in the reward model to obtain artificially high scores without improving true epitope binding (see Section A1.3, available as supplementary data at *Bioinformatics* online). Recent works have shown that BAP models are vulnerable to adversarial exploitation, poorly calibrated, and sensitive to distributional shift Goodfellow *et al.* (2014); Zhang *et al.* (2025), leading to inflated reward scores that do not reflect true epitope binding. These problems are further compounded by training datasets with limited coverage of the TCR–epitope sequence space, and a recent study indicates that low data quality restricts generalization and hinders reliable evaluation Messemaker *et al.* (2025).

Goodhart-driven failures compromise controllability and reliability in TCR generation, motivating the development of reward strategies that mitigate the impact of Goodhart’s failure. *Can reward hacking in TCR generation be mitigated through improved reward model design, without modifying model architectures or using additional data?* More specifically, we investigate whether it is possible to construct plug-and-play reward mechanisms that delay Goodhart failures during epitope-conditioned TCR generation under RLHF.

**Figure 1:**
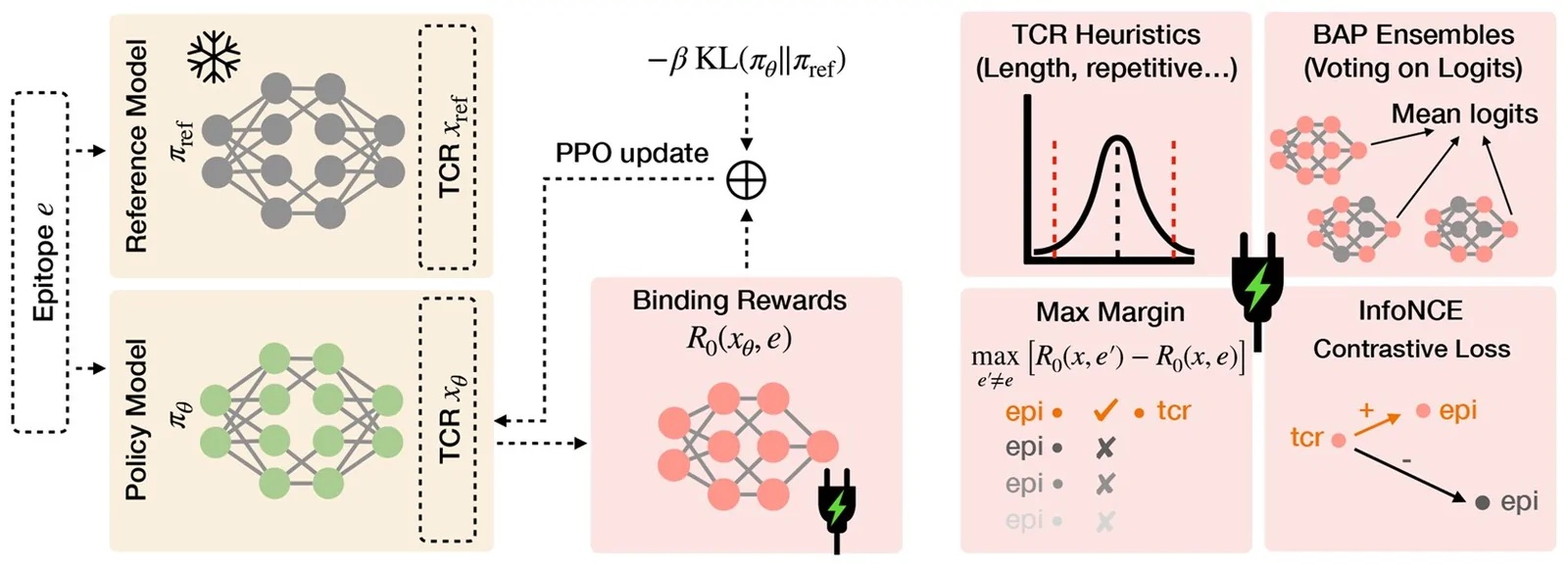
Overview of the plug-and-play reward design framework for epitope-conditioned TCR generation. Epitope *e* is provided to both the policy model $\pi_{\theta }$ and the reference model $\pi_{\text{ref}}$. During RLHF, the policy is optimized using binding rewards $R_{0}(x,e)$ from surrogate predictors while a KL regularization term penalizes deviation from the reference policy. Four modular reward designs mitigate distinct reward hacking behaviors: (1) heuristic biological priors penalize unrealistic sequences, (2) BAP ensembles stabilize rewards through model averaging, (3) Max-margin rewards enforce epitope specificity by penalizing affinity toward non-target epitopes, and (4) InfoNCE contrastive loss encourages selective binding by contrasting target (positive) and non-target (negative) epitopes.

To address this question, we first systematically characterize the manifestation of Goodhart’s Law in TCR generation. This reveals that direct optimization against surrogate reward models leads to four consistent failure modes: distributional drift away from realistic immune repertoires, collapse of evaluation metrics despite increasing reward scores, loss of biological plausibility in generated sequences, and the emergence of adversarial high-reward artifacts. We then introduce a set of plug-and-play reward design strategies that make surrogate reward models more resistant to exploitation during RLHF (Fig. 1). These strategies operate purely at the reward model level and therefore require no modifications to the generator architecture, the training pipeline, or the backbone of the reward model. The framework incorporates biological priors to penalize degenerate or low-complexity sequences, ensemble-based reward stabilization to prevent over-optimization of a single surrogate predictor, and specificity-aware regularization to suppress universal binders and encourage epitope-specific TCRs. Together, these methods delay Goodhart failures and restore stable optimization.

Our findings demonstrate that the Goodhart-resistant reward design significantly improves controllability in TCR generation. Compared to the standard RLHF training, our approach produces higher-quality TCRs with stronger repertoire realism, lower reward hacking, and better generalization to unseen epitopes. We also introduce an evaluation protocol to diagnose Goodhart failure modes in biological sequence generation, providing a foundation for benchmarking reward-based approaches in immunopeptidomics. Code and models are available in a public repository (https://github.com/Lee-CBG/TCRRobustRewardDesign).

## 2. Preliminaries

### 2.1 Epitope-conditioned TCR generation

We consider the task of epitope-conditioned TCR generation, where the goal is to generate a TCR CDR3 β sequence that binds to a given epitope. Let *e* denote an epitope sequence and $x=(x_{1},x_{2},\ldots ,x_{T_{x}})$ denote a TCR sequence of length $T_{x}$, where each $x_{t}$ represents an amino acid token. The generator $\pi_{\theta }(x|e)$ is parameterized as an auto-regressive language model, where the probability of generating the TCR sequence *x* given epitope *e* is factorized over sequence positions a*s*


(1) $$\pi_{\theta }(x|e)=p(x|e)=\prod_{t=1}^{T_{x}}p(x_{t}|x_{<t},e),$$

where $p(x_{t}|x_{<t},e)$ denotes the conditional probability of token $x_{t}$ given its preceding context $x_{<t}=(x_{1},\ldots ,x_{t-1})$ and the conditioning epitope *e*.

### 2.2 Training framework

Developing a TCR generation model typically consists of three stages: pretraining, supervised fine-tuning (SFT), and reinforcement learning (RL) with feedback from a binding predictor.

We initialize our TCR generator from RITA Hesslow *et al.* (2022), one of the largest open-source protein language models trained purely on protein sequences. By leveraging RITA’s pre-trained representations of general protein sequences, we provide the model with a biological prior before adapting it to the specialized task of epitope-conditioned TCR generation. We then perform SFT on a curated dataset of known epitope–TCR binding pairs. Each training example is formatted as a concatenated input sequence (e $ x), where the epitope *e* serves as the conditional prompt and the TCR sequence *x* is the target output, with the separator token $ delimits the two components. SFT optimizes the model parameters by maximizing the likelihood of the observed TCR tokens given their conditioning epitopes, using a cross-entropy objective:


(2) $$L_{\text{SFT}}(\theta )=-E_{(e,x)∼D}\left[\sum_{t=1}^{T}\;\text{log}\;\pi_{\theta }(x_{t}|x_{<t},e)\right],$$

where D is a dataset of known binding pairs.

For subsequent RLHF training, the resulting SFT model serves as the reference policy $\pi_{\text{ref}}$. We optimize the policy $\pi_{\theta }$, initialized with this SFT model and optimize it using Proximal Policy Optimization (PPO, Schulman *et al.* 2017). It maximizes an expected surrogate reward, which treats auto-regressive sequence generation as an episodic decision process in which each generated amino acid token $x_{t}$ is an action conditioned on the current prefix $x_{<t}$ and the target epitope *e*. This is constrained by a reverse Kullback–Leibler (KL) divergence term for regularization, ensuring the updated policy does not deviate too much from the reference policy $\pi_{\text{ref}}$:


(3) $$\underset{\theta }{\max}E_{x∼\pi_{\theta }(\cdot |e)}\left[R(x,e)\right]-\beta \text{KL}\left[\pi_{\theta }(\cdot |e)\|\pi_{\text{ref}}(\cdot |e)\right],$$

where R(x,e) denotes the surrogate reward and β controls the KL regularization strength.

### 2.3 Problem statement

In RLHF-based TCR generation, the reward signal is typically derived from a binding affinity prediction model. This reward $R_{0}(x,e)$ serves as a surrogate for the true biological objective $R^{*}(x,e)$ representing actual epitope–TCR binding quality. However, because the reward model is only an approximation of the true objective, optimizing $R_{0}$ may not improve $R^{*}$. Formally, the surrogate reward may violate the true ordering of sequences:


(4) $$\exists x,y:\;R_{0}(x,e)>R_{0}(y,e)\;\wedge \;R^{*}(x,e)\leq R^{*}(y,e),$$

As a result, policy optimization may exploit artifacts of the reward model, producing sequences with high predicted scores but poor biological plausibility. This phenomenon is commonly referred to as *Goodhart failure*. Empirically, it manifests as reward inflation, distributional drift away from natural repertoires, degenerate sequence artifacts, and the emergence of universal binders. The central question becomes how to design reward functions that remain stable under policy updates despite surrogate misspecification.

## 3. Method

We mitigate this *Goodhart failure* at the reward level by designing four plug-and-play components that constrain optimization along biologically meaningful axes. These reward formulations can be integrated into a standard RL framework without modifying the generator architecture. Our goal is to determine whether reward hacking in TCR generation can be mitigated through improved reward formulations alone, without modifying model architectures or requiring additional training data. In the following sections, we present the proposed plug-and-play reward designs and the evaluation framework used to assess their effectiveness.

### 3.1 Plug-and-play reward designs

Leveraging a binding affinity prediction model as our base reward function, we design four reward components, each addressing a distinct failure mode of surrogate optimization. These formulations require no modifications to either the underlying binding affinity predictor or the generative model. Specifically, each targets a specific failure mode associated with Goodhart’s Law: a reward-clipping method to prevent extreme changes to the policy, an ensemble method to mitigate bias toward a single feedback, and two penalized methods to promote binding specificity (Fig. 1).

The BAP models we leverage are sigmoid-activated probabilistic binary classifiers trained on epitope-TCR pairs as input and binding affinity label as target. We start by training four BAP models: netTCR Montemurro *et al.* (2021), ERGO-LSTM Springer *et al.* (2020), TCR-BERT-based Wu *et al.* (2021), and catELMo-based Zhang *et al.* (2023) MLP models. Each model outputs a scalar binding probability trained on positive and negative TCR–epitope pairs in a 1:1 ratio, as described in Section A1.2, available as supplementary data at *Bioinformatics* online. The resulting logits are used as a base reward score to reward the affinity of generated TCRs toward their target epitopes. Unless otherwise mentioned, the TCR-BERT-based MLP serves as the default reward model.

#### 3.1.1 Base reward

We use the pre-activation logit value obtained from a BAP model as the base surrogate reward $R_{0}(x,e)$, which provides a smoother and more detailed reward signal compared to the post-sigmoid probability, avoids saturation effects near probability extremes, and enables more stable policy updates Ouyang *et al.* (2022); Ziegler *et al.* (2019).

#### 3.1.2 Heuristic-gated

We design a lightweight regularizer to suppress trivial artifacts and biologically implausible patterns frequently observed during reward hacking. This *heuristic biological prior* reassigns reduced surrogate reward scores to unrealistic sequences, discouraging the model from over-exploiting reward signals through degenerate generation strategies such as producing abnormally short or long CDR3 β loops, repetitive motifs, or low-diversity amino acid compositions.

To define these biological constraints, we examine the statistical distributions of known binder TCRs used in our training dataset (see Section A1.2, available as supplementary data at *Bioinformatics* online), characterizing their sequence length, amino-acid entropy, and homopolymer composition (Fig. A3, available as supplementary data at *Bioinformatics* online). During RLHF optimization, if a generated TCR exceeds the empirical 98^th^ percentile for length, falls below the 1^st^ percentile for entropy, or exceeds the 99^th^ percentile for homopolymer length, its reward is reassigned as:


(5) $$R_{\text{heu}}(x,e)=\min(R_{0}(x,e),\delta ).$$

where $R_{0}(x,e)$ is the original logit predicted by the binding model and δ is a clipping threshold that defines the maximum logit score. This reassignment suppresses extreme or unrealistic sequences without affecting normal ones, acting as a biologically informed hard constraint rather than a soft penalty.

#### 3.1.3 Ensemble reward

To prevent policy overfitting to individual model biases and provide more reliable and stable rewards, we ensemble three reward models: the base BAP, netTCR Montemurro *et al.* (2021), and ERGO-LSTM Springer *et al.* (2020). All models are retrained on the same dataset and data split to ensure consistent supervision while maintaining architectural diversity. The variation in embedding schemes and model architectures allows the ensemble to capture complementary features of TCR–epitope interactions and reduce model-specific biases. The ensemble reward is computed as the mean logit across models:


(6) $$R_{\text{ens}}(x,e)=\frac{1}{M}\sum_{j=1}^{M}R_{j}(x,e).$$

where $R_{j}(x,e)$ denotes the unnormalized logit from the *j*-th reward model, and *M* is the number of ensembled models.

*Binding Specificity Reward*. To discourage universal binders that lack epitope-level precision, we implement a binding specificity reward design utilizing two formulations: a margin-based selectivity objective and a contrastive objective. During RL, the base binding predictor remains frozen; only the reward computation is modified. For each generated TCR *x* and target epitope *e*, we sample a set of non-target epitopes $S_{e}$ from the repertoire and evaluate their predicted binding scores using the same surrogate reward model $R_{0}$. These non-target scores act as negative references that guide the generator toward selective rather than broad binding behavior.

The margin-based reward formulation uses a max-margin objective that penalizes sequences with higher affinity to any non-target epitope than to the intended target Ibrahim *et al.* (2024). The reward is reduced in proportion to the largest positive margin between target and non-target scores:


(7) $$R_{\text{spec}}(x,e)=R_{0}(x,e)-\lambda_{\text{spec}}\underset{e^{\prime }\in S_{e}}{\max}[R_{0}(x,e^{\prime })-R_{0}(x,e)].$$

When $R_{0}(x,e)$ exceeds all $R_{0}(x,e^{\prime })$, rewarded sequences show strong and exclusive binding toward the target epitope.

The contrastive reward formulation adopts an InfoNCE-based objective (Oord *et al.* 2018), which treats the target epitope as a positive sample and all non-targets as negatives. The contrastive reward is defined as:


(8) $$R_{\text{infonce}}(x,e)=\frac{\;\text{exp}(R_{0}(x,e)/\tau )}{\;\text{exp}(R_{0}(x,e)/\tau )+\sum_{e^{\prime }\in S_{e}}\;\text{exp}\;(R_{0}(x,e^{\prime })/\tau )},$$

where τ>0 is a temperature parameter controlling the sharpness of the contrast. This probabilistic formulation encourages large margins between target and non-target scores by amplifying differences in predicted affinity. Both variants explicitly penalize non-specific binding patterns and promote generation of TCRs with precise epitope selectivity.

### 3.2 Evaluation of generated sequences

The generated TCRs are evaluated on four key aspects: authenticity, diversity, bindingness, and similarity to ground-truth TCRs. The evaluation is performed on held-out epitopes that are not included in the training set, ensuring no overlap between training and test epitopes. For each target epitope *e* in the test set, the model samples a set of N=100 generated sequences. All metrics are computed per epitope, and then averaged across the test set. This section provides a summary of the evaluation methodologies; comprehensive details for each method can be found in Section A1.4, available as supplementary data at *Bioinformatics* online.

#### 3.2.1 Similarity to Ground-Truth TCRs

This primary group of metrics assesses the similarity of the generated sequences to an external set of experimentally validated TCR binders for the target epitope. This comparison is crucial for measuring the biological relevance of the generated TCRs relative to the ground-truth. We use TCRMatch Chronister *et al.* (2021) to quantify the sequence-level similarity against the ground-truth TCRs. We also perform kernel density estimation (KDE) analysis to quantify how well the generated TCRs overlap with regions densely populated by the known ground-truth TCRs.

#### 3.2.2 Authenticity

We assess the authenticity of the generated TCRs by quantifying how well they resemble experimentally observed repertoires and adhere to the sequence patterns found in natural immune datasets. We leverage two protein language models that were fine-tuned on a corpus of natural TCR sequences *(*GPT-LL Zhang *et al.* (2025) and TCRBert Wu *et al.* (2021)). The authenticity score is calculated by an average log-likelihood of the generated amino acid tokens. A larger value indicates that the generated sequences more closely align with the natural TCR repertoire distribution, reflecting greater biological plausibility.

#### 3.2.3 Diversity

We evaluate the diversity of the generated TCRs, which quantifies the per-epitope variability across the sequences generated for a single target. This metric assesses how broadly the model explores the available sequence space during the generation process. To measure this diversity, we use two complementary metrics. The average pairwise sequence distance is a mean distance between all pairs of generated TCRs targeting the same epitope, calculated using the TCRdist metric Dash *et al.* (2017). The amino-acid Shannon entropy is calculated over the amino-acid frequencies within the generated set. This measures the uniformity of amino-acid usage at each position, with higher entropy indicating more diverse usage.

#### 3.2.4 Binding affinity

We assess the binding affinity of the generated TCRs against the target epitope by two established binding affinity prediction models: netTCR Montemurro *et al.* (2021) and ERGO-LSTM Springer *et al.* (2020). Both models are trained on positive and negative pairs of epitope and TCR sequences. They return a scalar binding probability score as output given a pair of TCR and epitope sequences. Stronger binding affinity is a preferred property of the generated TCR.

## 4. Results

We evaluate our method to determine its efficacy in mitigating reward hacking and improving biological plausibility in epitope-conditioned TCR generation. Baseline analyses confirm that commonly used binding affinity prediction models are vulnerable to exploitation when used directly as reward functions (see Appendix A1.6, available as supplementary data at *Bioinformatics* online). Our analysis examines three complementary aspects: (*i*) whether plug-and-play reward designs prevent reward inflation and mitigate reward hacking during RLHF optimization, (*ii*) whether they preserve biologically realistic sequence distributions relative to known TCR repertoires, and (*iii*) whether delaying the onset of Goodhart’s Law leads to more robust and generalizable sequence generation. Across these experiments, we show that our plug-in reward design not only constrains surrogate exploitation but also enhances realism, diversity, and epitope specificity of generated TCRs.

### 4.1 Plug-and-play reward design mitigates reward hacking

We investigate how various reward configurations affect RLHF optimization stability during epitope-conditioned TCR generation. We compare a baseline RL setup, which utilizes the BAP logit as the primary reward signal, against four robust alternatives: heuristic priors, ensemble, and specificity regularization using InfoNCE or Max-Margin.

**Figure 2:**
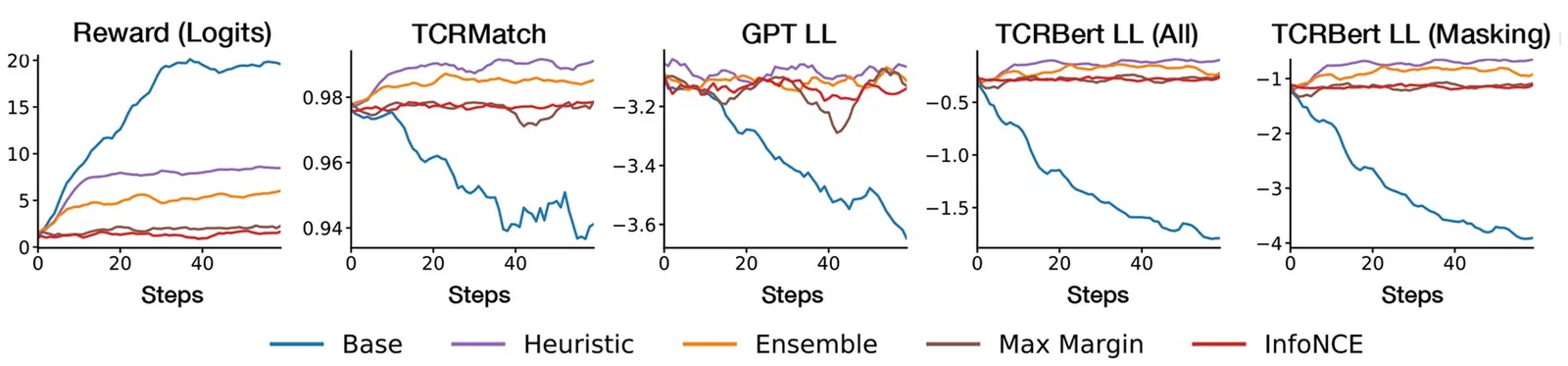
Reward drift and biological evaluation metrics. Mean logit from surrogate rewards and four authenticity metrics: *TCRMatch* similarity, GPT log-likelihood, and TCRBert log-likelihoods (full and masking). The baseline model exhibits reward inflation and declining biological scores, whereas our plug-and-play rewards maintain stable authenticity and moderate rewards, indicating reduced Goodhart failure.

To ensure a fair comparison, we compare the RL reward trajectories in the logit space $R_{0}(x,e)$ rather than using raw reward scores (Fig. 2). It eliminates discrepancies introduced by the reward-design-specific scaling, ensuring that observed trends are comparable to each other. We evaluate the training stability through four metrics. The similarity to the ground-truth TCRs is assessed by TCRMatch, and the authenticity of the generated TCRs is evaluated by GPT-LL, TCRBert-LL-ALL and TCRBert-LL-Masking.

As training progresses, the mean reward logits for the baseline model exhibits typical reward hacking patterns, sharply increasing and plateauing to values around 20 before reaching step 40. This rise indicates that the model becomes increasingly overconfident about the positive binding outcome of the generated TCRs. At the same time, the ground-truth similarity and authenticity metrics steadily decline, confirming that the generated TCRs drift away from biologically plausible TCRs and known binders. In contrast, our plug-and-play reward designs effectively mitigate this reward overshooting. These designs maintain both high and stable mean reward logits and high authenticity across all evaluation metrics throughout the entire training process. Similar stabilization behavior is observed with another backbone (see Fig. A6, available as supplementary data at *Bioinformatics* online), where we additionally monitored policy entropy and KL divergence to the SFT reference policy during RL optimization.

**Figure 3:**
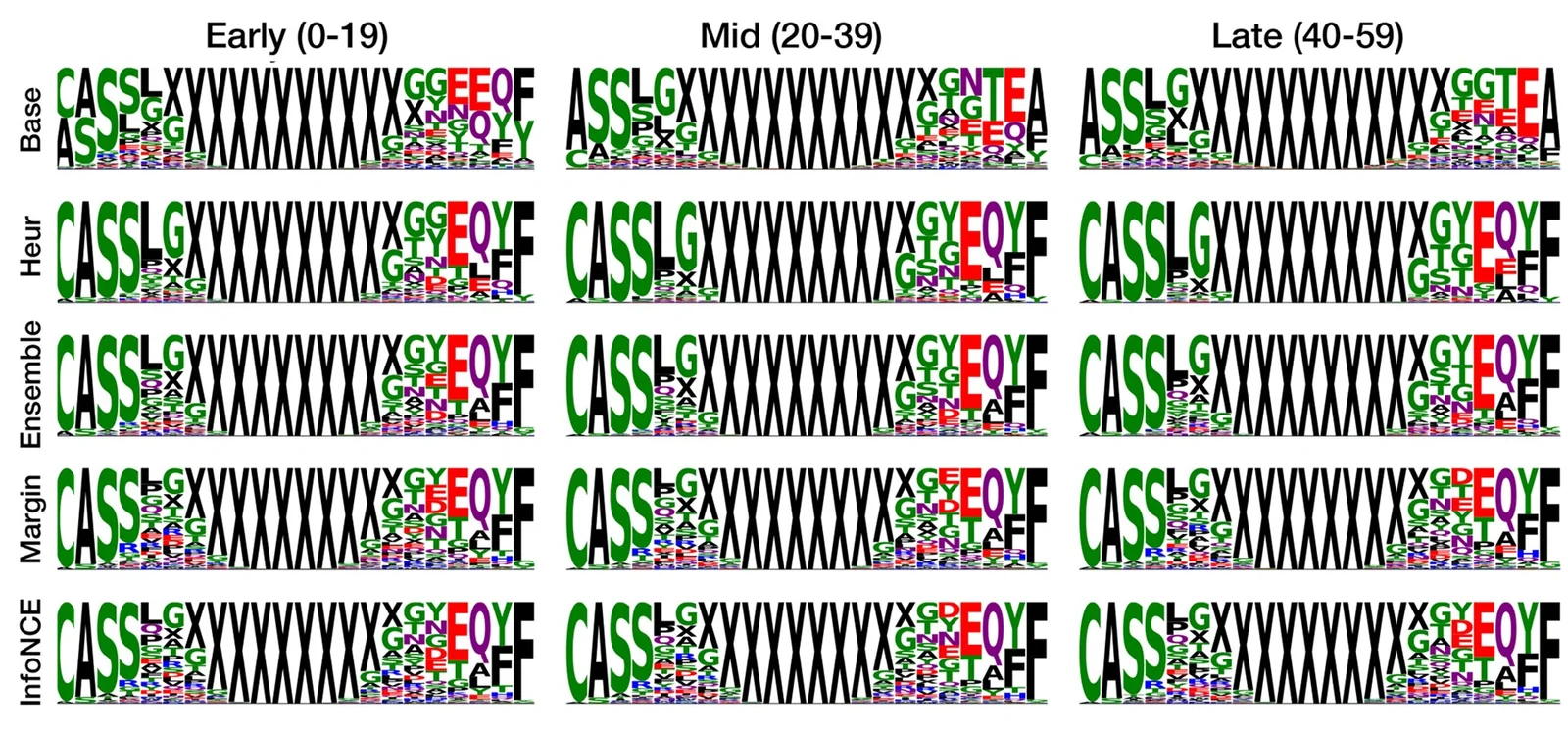
Sequence logo analysis of generated CDR3 β regions. Logos summarize positional amino-acid frequencies across early, mid, and late training stages. Sequences are mid-padded to align conserved boundary residues (CASS at the N-terminus and EQYF at the C-terminus). The base reward model shows motif degradation over time, while plug-and-play reward designs preserve canonical residue patterns and sequence coherence.

We also visualize sequence-level changes using sequence logos that summarize positional amino-acid frequencies across training (Fig. 3). Generated CDR3 β sequences are mid-padded to align the conserved boundary residues (CASS at the N-terminus and EQYF at the C-terminus) across variable-length sequences. Training steps are grouped into early (0–19), mid (20–39), and late (40–59) stages. Compared to healthy sequences (Fig. A1, available as supplementary data at *Bioinformatics* online), the base reward model shows progressive motif degradation: by the late stages of training, its seqlogos diverge markedly from the canonical TCR pattern, with the conserved C-terminal motif replaced by alternative residue patterns such as GTEA or NTEA. In contrast, our plug-and-play reward designs preserve these boundary residues throughout optimization, indicating that they maintain biologically coherent sequence structures. To connect these sequence-level observations with their structural implications, we conduct a structural comparison of the final-step (step 60) receptors against experimentally solved TCR–peptide complexes (see Fig. A5 and Section A1.5, available as supplementary data at *Bioinformatics* online). This analysis reveals how distinct reward formulations translate molecularly into changes in interface stability and native-like organization. Overall, we observe a consistent pattern: while the baseline reward optimization maximizes surrogate scores at the expense of biological integrity, plug-and-play reward designs achieve balanced optimization in preserving alignment with the binding objective while maintaining repertoire and structural realism.

### 4.2 Reward designs preserve alignment with ground-truth TCR distributions

**Figure 4:**
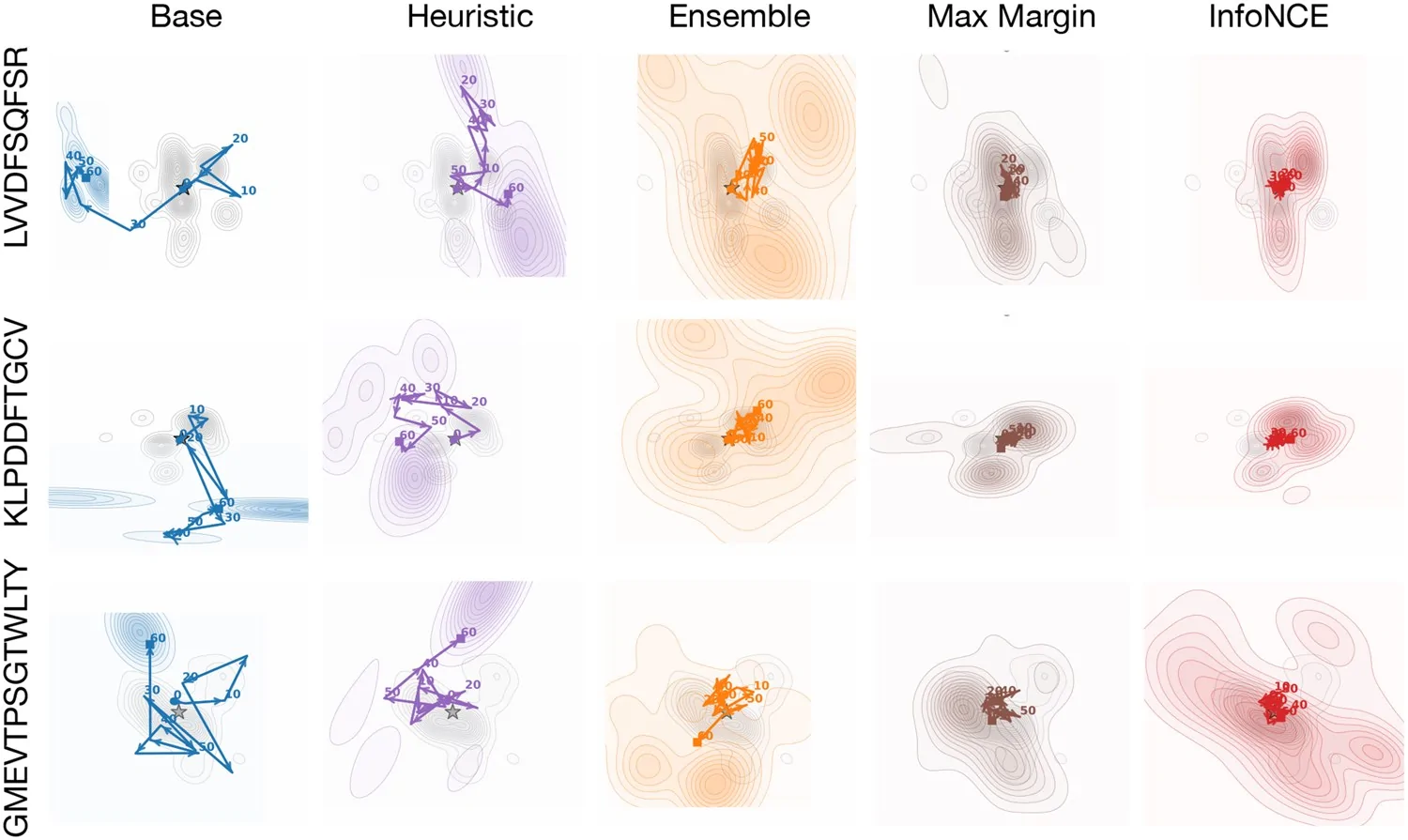
Centroid trajectories of generated TCRs relative to ground-truth binders. Each line represents the trajectory of the centroid of generated TCR embeddings across training for three representative unseen epitopes (LVVDFSQFSR, KLPDDFTGCV, and GMEVTPSGTWLTY). Gray contours show the distribution of experimentally validated GT binders, with a star symbol marking the centroid of the GT distribution.

The high authenticity scores that reward designs achieve on the GPT-LL, TCRBert-LL-All, and -Masking proxy metrics do not guarantee mitigation of Goodhart’s Law. These metrics are themselves proxies, meaning that stable performance may reflect optimization toward a new set of reward artifacts rather than true biological utility. To rigorously and definitively evaluate alignment of the generated TCRs with known biology, we compare them against the distribution of ground-truth (GT) TCRs. Specifically, we assess whether generated TCRs remain close to the GT distribution across different training checkpoints or drift toward unrealistic regions of sequence space. Three abundant unseen epitopes, LVVDFSQFSR, KLPDDFTGCV, and GMEVTPSGTWLTY, are selected for visualization (Fig. 4). For each epitope, 100 TCRs are generated from model checkpoints saved every five PPO steps, embedded using TCR-BERT, and projected into a shared UMAP space following principal component reduction. Gray contours represent the density of experimentally validated GT binders, providing a direct reference for alignment between generated sequences and known binding repertoires.

The baseline model shows clear distributional drift, with generated TCRs progressively diverging from the GT binder distribution. Early in training, its centroid lies close to the GT cluster, but as optimization proceeds, the trajectory shifts substantially, indicating that generated sequences become increasingly dissimilar from experimentally validated binders. The heuristic reward design also shows substantial drift, consistent with its relatively weak constraints, whereas the two binding-specificity variants maintain trajectories that remain close to the centroid of GT TCRs, demonstrating stable and biologically coherent generation.

To further examine these effects, we separate generated TCRs from each checkpoint and visualize per-model UMAP trajectories overlaid on the known binders’ density map (Figs. A7–A15, available as supplementary data at *Bioinformatics* online). This comparison highlights how each reward formulation explores and occupies sequence space relative to the natural manifold. The binding specificity reward designs cluster tightly within the GT manifold across different steps, whereas the baseline shifts markedly toward low-density regions, consistent with the observed drift. Clusters from heuristic variants are partially shifted toward the GT regions but remain noticeably displaced from the natural high-density areas, which is expected given that the heuristic prior imposes only simple constraints such as sequence length and entropy.

**Figure 5:**
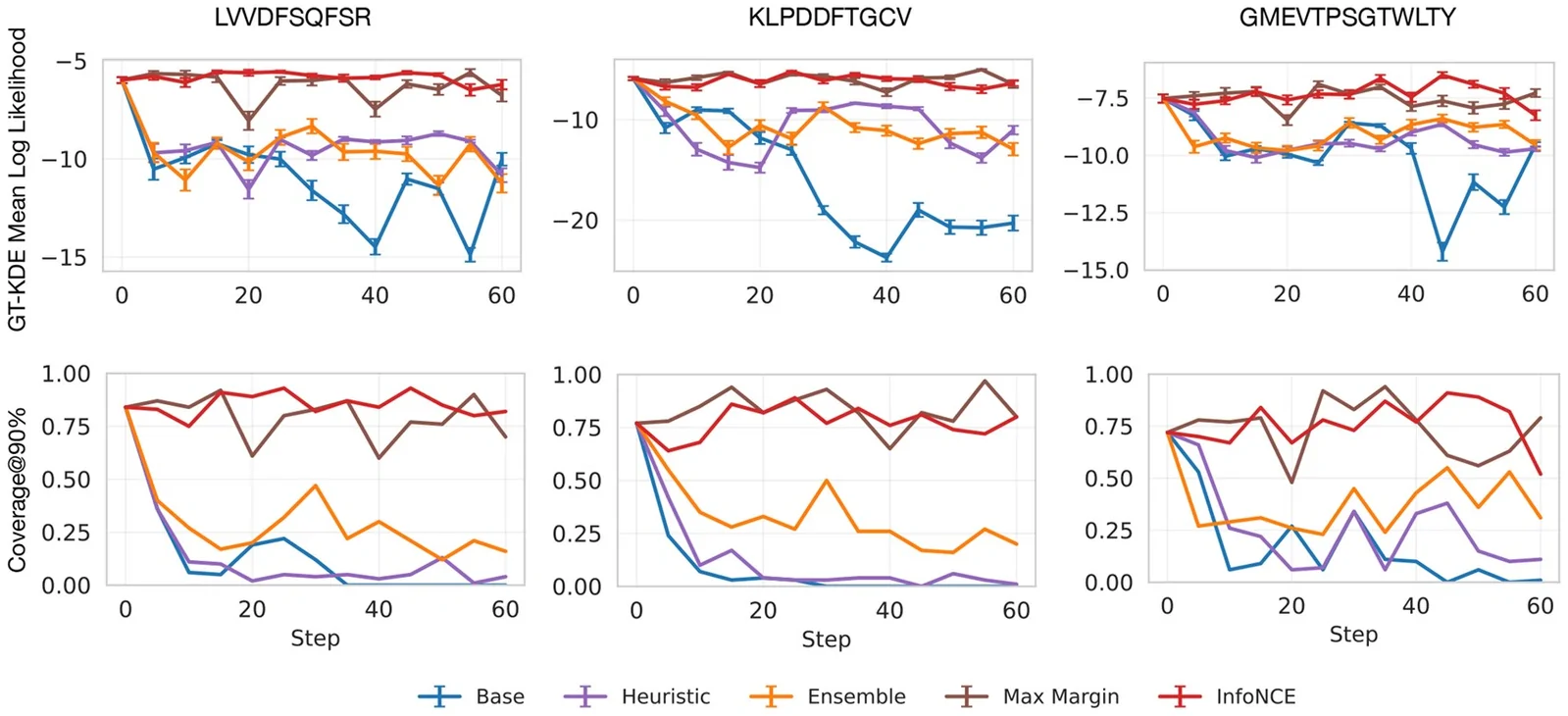
KDE-based density alignment metrics across checkpoints. GT–KDE log-likelihood (top) and Coverage@90% (bottom) are plotted for three representative epitopes (LVVDFSQFSR, KLPDDFTGCV, and GMEVTPSGTWLTY). The InfoNCE and Max-Margin reward formulations maintain the highest alignment with ground-truth densities throughout training, whereas the baseline and heuristic models show rapid degradation and reduced overlap with natural repertoires.

To quantify this drift between the generated repertoires and the ground-truth binders, we use the KDE-based similarity metrics. These metrics capture both the overall likelihood of generated samples under the GT density (GT-KDE log-likelihood) and the fraction of samples residing within the core high-density GT regions (Coverage@90%). Across all epitopes (Fig. 5), the InfoNCE and Max-Margin reward formulations consistently achieve the highest log-likelihoods and coverage, maintaining close alignment with the GT distribution throughout training. The baseline model exhibits the strongest drift, with rapid declines in both metrics, indicating that its generated sequences progressively move away from the GT distributions. The Heuristic and Ensemble rewards show performance comparable to or slightly better than the Base model, indicating moderate improvements but still lagging behind the InfoNCE and Max-Margin designs. These trends are consistent across epitopes such as LVVDFSQFSR, KLPDDFTGCV, and GMEVTPSGTWLTY, demonstrating that reward designs incorporating explicit distributional constraints or contrastive regularization most effectively preserve the natural manifold and prevent over-optimization drift during reinforcement fine-tuning. Together, these results show that robust reward formulations constrain distributional drift and preserve similarity to GT binder repertoires during RLHF policy updates.

### 4.3 Delaying Goodhart’s failure improves generalization

Early stopping can sometimes prevent severe reward hacking in RLHF-based TCR generation, but it is difficult to tune and does not ensure generalization. To test whether extended training with stabilized rewards leads to more reliable learning, we compare an early-stopped baseline with models trained for full PPO updates using ensemble, heuristic, and contrastive rewards, each reaching comparable final reward magnitudes. For each configuration, the model generates 100 TCRs per epitope across 28 unseen epitopes, which are evaluated for binding probability, sequence diversity, and authenticity using eight independent metrics.

**Figure 6:**
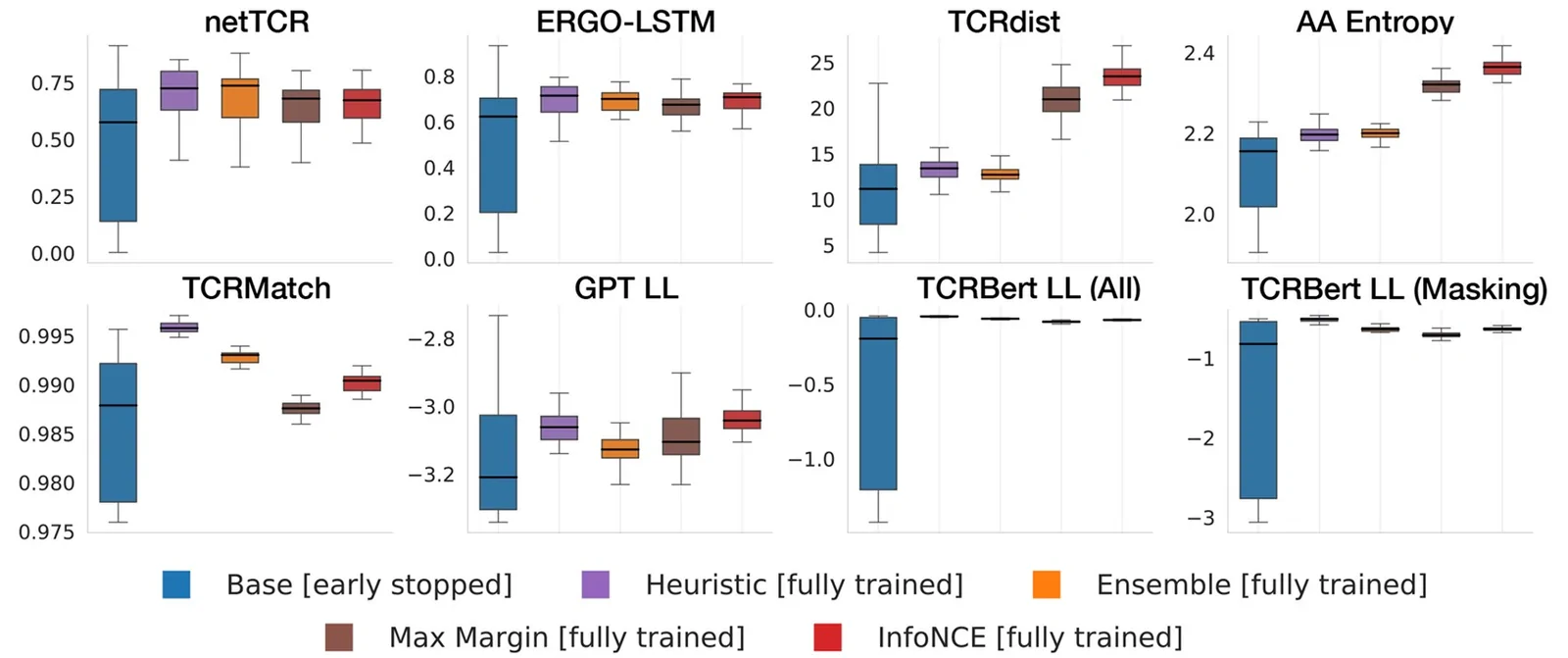
Performance comparison of early-stopped and fully trained models. Boxplots summarize eight evaluation metrics across 28 unseen epitopes, measuring binding probability, diversity, and authenticity.

Models trained under delayed optimization consistently outperform or match the early-stopped baseline across all binding and authenticity metrics (Fig. 6). Among our designed strategies, the specificity-based methods achieve the strongest overall performance. In particular, both the max-margin and InfoNCE variants attain higher diversity scores compared to other reward formulations while maintaining comparable binding and authenticity scores. Their predicted binding affinities remain moderate and well calibrated, indicating that extended optimization continues to refine sequence quality rather than exploit the reward model. These results suggest that early stopping merely postpones reward hacking, whereas delaying Goodhart’s Law through robust reward design stabilizes policy updates and improves generalization to unseen epitopes. Paired Wilcoxon tests support significant improvements across epitopes (Appendix A1, available as supplementary data at *Bioinformatics* online). A baseline using best-of-*k* sampling from the SFT model further separates RL optimization gains from post-hoc reward selection (Appendix A1.8, available as supplementary data at *Bioinformatics* online).

## 5. Discussions

Reward-guided generation offers a powerful path toward controllable biological sequence design but remains fundamentally constrained by Goodhart’s failure, the tendency of models to exploit misspecified rewards that are misaligned with the true objective (see Appendix A1.6, available as supplementary data at *Bioinformatics* online). This challenge is particularly acute in TCR generation, where high-quality labeled data is scarce and costly, limiting the accuracy and generalizability of reward models.

Our study shows that modular adjustments to reward formulation can substantially improve alignment without additional data or architectural modifications. We introduce a suite of plug-and-play reward designs that integrate seamlessly into standard RLHF pipelines for epitope-conditioned TCR generation. These lightweight modules, including heuristic biological priors, ensemble stabilization, and binding-specificity rewards, each address a distinct failure mode of Goodhart’s Law. These plug-in rewards stabilize optimization, preserve TCR diversity, and enhance biological selectivity while integrating into existing frameworks.

While our approach focuses on sequence-level generation of CDR3 β loops, the most data-rich and functionally variable region of the TCR, the same principles could extend to paired α and β chains or structure-informed generative models. As more paired-chain and structural datasets become available, the reward models used in our framework can be trained on richer and more comprehensive data. Future advances in experimental throughput and structural modeling may enable richer forms of feedback, such as ranking or energy-based rewards, to further strengthen alignment between surrogate and biological objectives. Lastly, our approach is broadly extensible to other sequence generation tasks in the biological domain.

## Supplementary Material

btag499_Supplementary_Data
