# Supplementary material for: Mitigating Goodhart’s law in epitope-conditioned TCR generation using plug-and-play reward designs
Source: Bioinformatics. 2026 Aug 21;42(Suppl 2):btag499. doi: 10.1093/bioinformatics/btag499 (PMC13501281; doi:10.1093/bioinformatics/btag499)
Supplement: Supplementary file 1 [file btag499_supplementary_data.pdf]

A1 Supplementary Information

A1.1 Related Works

Epitope-conditioned TCR Generation. Prior works have developed epitope-conditioned TCR generative models, which generate TCRs targeting a specified epitope. Experiments show that epitope-conditioned TCR generation yields TCRs that are more likely to bind to the target epitope compared to unconditioned models [10, 18, 28, 30, 32]. Crucially, these models do not take advantage of additional reward signals available through binding datasets or binding affinity prediction models, leaving additional supervision through controlled generation on the table.

Controlled TCR Generation with Reward Functions. In order to further improve the quality of generated sequences, prior works have employed RL for post-training [2, 22, 25, 31] or used a reward function for post-hoc steering [27]. These methods incorporate the additional supervision provided by binding datasets or binding prediction models by steering the generative model to generate high-binding-affinity TCRs.

Reward Hacking in RL and Goodhart's Law. Goodhart's Law in machine learning broadly states that when a proxy to a goal is used as an optimization objective, the proxy fails to remain an effective proxy of the goal [8, 15]. This manifests in RL optimization as a problem known as reward hacking, where the RL process employs a reward model that serves as a proxy to some objective. Using RL with such a reward can cause the policy model to exploit shortcuts in the reward model, leading to unintended consequences as a result of reward misspecification. Reward hacking has been widely observed across different applications of RL, including toy problems, robotics, and language modeling [11, 13, 5]. This includes protein language modeling, where other works identify reward hacking, attributed to reward misspecification, while steering generative models using RL [2, 22].

A1.2 Data

A total of 150,008 known epitope–TCR binding pairs were collected from the three publicly available databases: VDJdb [20], IEDB [24], and McPAS [23]. The combined dataset includes 982 unique epitopes and 140,675 unique TCRs. For supervised fine-tuning of the TCR generator, we excluded epitopes with fewer than 100 known binding TCRs to ensure sufficient representation. The remaining epitopes were randomly split into training and test sets based on epitope identity, ensuring that no epitope appears in both partitions. For reinforcement learning, only epitopes are used as input prompts; no known binder TCRs are required during policy optimization. We maintained the same training–testing epitope split as in the supervised stage to prevent data leakage. Binding affinity prediction models used as reward models were trained using both positive and negative samples. Negative samples were generated by pairing epitopes with TCRs drawn from healthy background repertoires [7, 29], maintaining a 1:1 ratio with positive pairs and split into training and test sets.

A1.3 Goodhart's Law

Goodhart's Law [8] states that "when measure becomes a target, it ceases to be a good measure." In the context of RL, this principle directly explains the phenomenon of reward hacking frequently observed when a surrogate reward is used to approximate the complex true objective. Consequently, the policy will inevitably find ways to exploit in the surrogate reward rather than achieving the true objective.

Ideally, $R_0(x, e)$ should accurately approximate the true objective $R^*(x, e)$. However, optimization against imperfect surrogate is highly vulnerable to *Goodhart failure*, where maximizing R_0 no longer improves the true objective R^*. This misalignment can be formalized as an order violation:

$$\exists\, x, y :\ R_0(x, e) > R_0(y, e)\ \wedge\ R^*(x, e) \leq R^*(y, e), \tag{A1}$$

and as policy degradation under surrogate optimization:

$$\theta' = \arg\max_{\theta} \mathbb{E}_{x \sim \pi_\theta}[R_0(x, e)] \ \Rightarrow\ \mathbb{E}_{x \sim \pi_{\theta'}}[R^*(x, e)] \ \leq\ \mathbb{E}_{x \sim \pi_\theta}[R^*(x, e)]. \tag{A2}$$

Consequently,

$$\arg\max_x R_0(x,e) \neq \arg\max_x R^*(x,e), \tag{A3}$$

indicating that the policy optimized against R_0 may exploit artifacts of the surrogate model rather than improving generation quality. This misalignment is exacerbated in TCR sequence generation, where training data are limited and surrogate predictors generalize poorly beyond their training distribution.

A1.4 Evaluation of Generated Sequences

A1.4.1 Similarity to Ground-Truth TCRs

TCRMatch. TCRMatch [3] quantifies sequence level similarity between each generated TCR x and a set of experimentally validated binders $\mathcal{B}_e$ for the target epitope e. Specifically, the overall score is defined as follows.

$$\mathrm{Sim}(x,e) = \max_{y\in\mathcal{B}_e} \sigma(x,y), \tag{A4}$$

where $\sigma(x,y)$ is the TCRMatch similarity score. TCRMatch is a k-mer–based matching algorithm that evaluates the similarity between two TCR sequences by gradually decomposing them into k-mers (subsequences of length k). The final score is obtained by summing the similarity contributions across all k-mer sizes and normalizing the result. Higher similarity indicates that the generated receptor shares repertoire level features with known epitope specific TCRs, suggesting stronger biological plausibility.

KDE-Based Similarity Metrics. To evaluate distributional alignment between generated TCRs and GT repertoires in embedding space, we apply a Gaussian kernel density estimator (KDE) to the embeddings of experimentally validated GT binders for each epitope. The fitted KDE provides a continuous estimate of the GT density, enabling two complementary metrics: (i) the GT-KDE log-likelihood, defined as the average log-probability of generated samples under the GT density, and (ii) the Coverage@90%, defined as the fraction of generated TCRs located within the top 90% highest-density region of the GT distribution. These KDE-based metrics quantify how well generated sequences overlap with regions densely populated by experimentally validated TCRs, with higher values indicating stronger alignment and closer similarity to GT binders.

A1.4.2 Authenticity

GPT-LL. We measure the repertoire authenticity of each generated TCR using a language model π_{rep} we trained on natural TCR repertoires. The model π_{rep} is initialized from pretrained protein language model weights and fine-tuned on a corpus of natural CDR3β sequences to capture authentic sequence patterns and amino acid dependencies [9, 31]. For a generated sequence $x = (x_1, \ldots, x_T)$ of length T, the normalized token-level log-likelihood (average log-likelihood per residue) are computed as

$$\mathrm{LL}_{\mathrm{rep}}(x \mid e) = \frac{1}{T}\sum_{t=1}^{T} \log P_{\pi_{\mathrm{rep}}}\big(x_t \mid x_{<t}, e\big), \tag{A5}$$

where $P_{\pi_{\mathrm{rep}}}(x_t \mid x_{<t}, e)$ denotes the softmax probability of token x_t predicted by the model given the preceding context and the conditioning epitope e. Normalizing by sequence length ensures comparability across CDR3s of different lengths.

BERT-LL. We also use TCR-BERT [26], a version of the BERT masked language model [6] pretrained on TCR sequences, to evaluate the authenticity of generated TCRs. Unlike GPT-style autoregressive models, TCR-BERT predicts masked amino acids using bidirectional context, providing a complementary assessment of repertoire plausibility. Given a generated TCR sequence $x = (x_1, \ldots, x_T)$, the model outputs token-wise logits over the amino-acid vocabulary V, which are converted into probabilities using a softmax function.

We compute two authenticity scores: full-sequence log-likelihood and masking pseudo-log-likelihood:

$$\mathrm{LL}_{\mathrm{full}}(x;\theta) = \frac{1}{T}\sum_{t=1}^{T} \log P_\theta(x_t \mid x_{1:T}), \tag{A6}$$

$$\mathrm{LL}_{\mathrm{mask}}(x;\theta) = \frac{1}{T}\sum_{t=1}^{T} \log P_\theta(x_t \mid x_{\setminus t}), \tag{A7}$$

where $P_\theta(x_t \mid x_{1:T})$ and $P_\theta(x_t \mid x_{\setminus t})$ denote the probabilities of residue x_t given the full sequence context or the sequence with the tth residue masked, respectively.

Another complementary authenticity measure is the generation probability, or p_{gen} [19], of a TCR sequence under V(D)J recombination models. Such measures are widely used to quantify how likely a receptor sequence is to arise from the generative process of immune-repertoire formation. We did not use p_{gen} as a primary evaluation metric because our task is epitope-conditioned CDR3β generation, and the main failure mode studied here is reward-induced loss of target-specific plausibility rather than repertoire-generation likelihood alone. A high-p_{gen} sequence is not necessarily specific to the target epitope, while a low-p_{gen} sequence may still be biologically valid after antigen-driven selection. Thus, p_{gen} is complementary to the language-model likelihood, ground-truth binder alignment, KDE distributional alignment, and diversity metrics used in this work. Incorporating p_{gen} into future benchmarks would further strengthen evaluation of reward-guided TCR generation.

A1.4.3 Diversity

TCRdist. We compute pairwise distances between CDR3β sequences using a simplified implementation of TCRdist [4]. Sequences are aligned using an IMGT-style mid-padding scheme to ensure consistent sequence lengths, and amino-acid differences and gaps are penalized according to substitution-based distance rules consistent with the original TCRdist formulation. The mean pairwise distance across all sequence pairs within each epitope-specific set X_e provides a per-epitope diversity score:

$$\overline{D}_{\mathrm{TCR}}(e) = \mathrm{mean}\big(d(x_i, x_j)\big), \quad \forall\, x_i, x_j \in X_e,\, i < j, \tag{A8}$$

where $d(x_i, x_j)$ denotes the substitution-based sequence distance. Larger $\overline{D}_{\mathrm{TCR}}$ values indicate greater sequence variability among generated TCRs within each training checkpoint.

Amino-acid entropy. To quantify compositional diversity, we compute the Shannon entropy over amino-acid frequencies within the generated set. For each epitope e, the empirical frequency $p(a)$ of residue a is estimated from all generated sequences X_e, and the entropy is calculated as

$$H_e = -\sum_{a \in \mathcal{V}} p(a) \log_e p(a), \tag{A9}$$

Both metrics are computed after randomly subsampling up to 100 sequences per epitope to ensure comparability across epitopes of varying data sizes. Higher $\overline{D}_{\mathrm{TCR}}$ and H_e values indicate more diverse and less collapsed repertoires, whereas lower values suggest redundancy or convergence toward a small number of sequence motifs.

A1.4.4 Binding Affinity

To estimate the binding probability between generated TCRs and their target epitopes, we employ two established binding predictors: netTCR [17], a convolutional neural network (CNN)–based model that captures local residue patterns via convolutions, and ERGO-LSTM [21], a bidirectional LSTM architecture designed to capture long-range sequential dependencies. Both models are trained on a balanced (1:1) dataset of positive and negative pairs. See Section A1.2 for details about the training dataset. They return a scalar binding probability score as output given a pair of TCR and epitope sequences.

A1.5 Structural Analysis

A1.5.1 Method

Structural models of generated TCR CDR3β regions predicted to bind to the influenza epitope GILGFVFTL were predicted using AlphaFold3 [1]. Structural context was approximated using HLA-A*02:01-restricted $\alpha\beta$ TCRs known to recognize the same peptide (PDB IDs: 1OGA, 2VLJ, 2VLK, 2VLR, 5E6I, 5EUO, 5ISZ, 5TEZ). For each group and model step, a generated CDR3 sequence β was randomly selected to represent group-level behavior due to computational limits. Each generated CDR3β was substituted into its corresponding reference complex while all other chains were retained. Sequence alignment and loop substitution were guided by global alignments using TCRdist penalty parameters specific to the CDR3β region [4]. Structural modeling was performed using ASU's AlphaFold3 Apptainer (v3.0.0). Reference complexes were computed first to cache multiple sequence alignments (MSAs); these cached MSAs were reused only for unmodified chains in later predictions, ensuring that altered β-chains were inferred independently. All reference structures were reproduced within 2 Å RMSD of their deposited geometries. All predicted complexes were refined using Rosetta [14] Relax (v3.7.1) to remove steric clashes and improve local backbone geometry, and binding metrics were computed from relaxed structures using Rosetta's Interface Analyzer (v3.7.1). Models were visualized and inspected in ChimeraX [16] (v1.10rc).

A1.5.2 Results

To contextualize the structural behavior of the generated receptors, we compared step-60 predictions across all design strategies to the two experimentally solved GILGFVFTL-specific TCRs most frequently recovered in our alignment analysis: 1OGA and 5ISZ (Fig. A5). Both structures exhibit stable and well-organized TCR–peptide interfaces. The Base model showed the weakest convergence toward these native interaction profiles: by step 60 it retained only a minimal TCR–peptide contact and exhibited the poorest quantitative interface metrics (iPTM = 0.55, $\Delta G = -36.4$ kJ/mol, buried hydrophobic area = 1054 Å^2, TCR–pMHC hydrogen bonds = 7). The Heuristic model demonstrated moderate improvement, increasing interface hydrogen bonds (6→10) and hydrophobic burial (951→1162 Å^2), with a modest gain in predicted binding affinity ($\Delta G = -39.1$ kJ/mol). Ensemble produced more substantial refinement, achieving higher hydrophobic burial (1355 Å^2), increased hydrogen bonding (9), and improved ΔG (-46.0 kJ/mol). InfoNCE showed strong and consistent optimization, attaining favorable binding free energy ($\Delta G = -53.0$ kJ/mol) and balanced interface organization (1206 Å^2 burial, 8 hydrogen bonds). Among all paradigms, Max-Margin achieved the most native-like and energetically stable interfaces at step 60, with the strongest predicted affinity ($\Delta G = -62.6$ kJ/mol), highest iPTM (0.87), substantial hydrophobic burial (1282 Å^2), and a robust interfacial hydrogen-bond network (11). Collectively, these results indicate that while the base model fails to approximate biologically realistic binding behavior, Max-Margin and InfoNCE reliably converge toward more coherent and native-like TCR–epitope interaction geometries, consistent with experimentally validated structures such as 1OGA and 5ISZ. Note that GILGFVFTL was the only evaluated epitope for which we identified suitable experimentally solved TCR–pMHC structures (HLA*02:01-restricted and bound to TCR) with known binders in PDB. In addition, our model generates only CDR3β sequences, whereas reliable TCR–pMHC structural modeling depends on paired $\alpha\beta$ chains, CDR1/2/3 loops, peptide, and MHC context.

A1.6 Vulnerability of Binding Affinity Predictors to Reward Hacking

In this section, we demonstrate BAP models are vulnerable to reward hacking. We first show that the observed high volume of false positives in a corpus of randomly generated sequences serves as a primary indicator of a potential reward misspecification, which can enable downstream reward hacking. In addition to the positive and negative data splits used for BAP training, we generate a 3rd split of fake data containing randomized amino acid mutations so that the Shannon entropy of the sequence matches a specified value using simulated annealing (SA) [12]. Specifically, for the 150,008 sequences in this split, we vary the target entropy to be uniformly distributed between $[\mu, \mu - 4\sigma]$, where $\mu \approx 2.2611$ and $\sigma \approx 0.1453$ are the mean and standard deviation of the amino acid entropy of the healthy TCR repertoire. We compute logits for all three splits using the pretrained BAP models, showing that the models, particularly non-BLOSUM-based ones, assign high logit values to the fake data compared to the positive data (Fig. A2A). We use the TCR-BERT

MLP for downstream tasks due to its smoother logit distribution compared to netTCR and ERGO along with faster inference speed and less extreme reward hacking compared to catELMo.

We also use SA to directly attack the BAP models. We set the target epitope to the influenza A peptide GILGFVFTL, set the target logit value to an extremely high value, and initialize a population of candidate sequences to a sample of healthy TCRs. We then use random mutations to mutate the candidate population and calculate the logits using the target BAP model for the SA algorithm to optimize. We also prune candidate sequences with logit values in the bottom 50% when the logit distribution's standard deviation is above a threshold to accelerate convergence. We observe that the algorithm generates visibly abnormal sequences with extreme logit values, with catELMo- and TCR-BERT-based MLP models showing more extreme values and sequences compared to the BLOSUM-based ERGO and netTCR models (Fig. A2B–C).

We then demonstrate reward hacking in a baseline RL post-training setup, where we use both a TCR-BERT MLP and a catELMo MLP as our base reward models to steer the epitope-conditioned TCR generation model. Here, we do not use any reward hacking mitigations besides the standard KL regularization term, allowing the policy to overfit to the vulnerable BAP models. We use KL divergence to the SFT reference policy as a direct measure of policy drift during PPO optimization. In the baseline setting, increasing reward is accompanied by increasing KL divergence and decreasing policy entropy, indicating that the policy moves away from the reference distribution while collapsing toward reward-exploiting sequences. In contrast, the robust reward designs show more bounded KL trajectories, supporting the interpretation that they mitigate Goodhart failure by constraining over-optimization of surrogate-model artifacts. As shown in Fig. A4, the policies trained with baseline reward formulations quickly exhibits classical signs of reward hacking: the mean reward escalates sharply over training, accompanied by increasing reward variance, a steep rising in KL divergence, and a collapse in entropy. These patterns indicate a classic Goodhart failure: the policy over-optimizes the surrogate reward, diverging from the reference distribution and generating increasingly degenerate sequences.

A1.7 Hyperparameters and Reproducibility Details

We report the main hyperparameters used for PPO fine-tuning, reward construction, sequence generation, and evaluation. The SFT generator was initialized from a local supervised fine-tuning checkpoint. PPO fine-tuning used a learning rate of 1.41×10^{-5}, batch size of 128, mini-batch size of 32, PPO clipping coefficient of 0.2, entropy coefficient of 0, value-function coefficient of 0.1, and 60 PPO update steps. The KL coefficient was set to 0.5.

Sequence generation used sampling temperature 1.0, top-$k = 0$, top-$p = 1.0$, and a maximum generation length of 42 new tokens. The base reward was the pre-sigmoid logit from the TCRBert-based binding affinity predictor. The Heuristic-Gated reward used empirical filtering thresholds of CDR3β length 9–21, amino-acid entropy at least 1.76, and maximum homopolymer length at most 3, with clipping threshold $\delta = 10.0$. The Ensemble reward averaged logits from TCRBert, a netTCR-style CNN, and ERGO-LSTM. The Max-Margin reward used 4 sampled non-target epitopes with specificity weight $\lambda_{\text{spec}} = 1$, while the InfoNCE reward used temperature $\tau = 0.25$ with the same non-target epitope sampling strategy.

For evaluation, each model generated 100 TCRs per held-out epitope across 28 unseen epitopes. All metrics were computed per epitope and then summarized across epitopes. Binding probability was evaluated using netTCR and ERGO-LSTM. Diversity was measured by average pairwise TCRdist and amino-acid Shannon entropy. Authenticity was evaluated using GPT-style repertoire log-likelihood and TCR-BERT log-likelihood. Similarity to experimentally validated binders was measured using TCRMatch and KDE-based density alignment metrics.

A1.8 Post-hoc Best-of-k Sampling of the SFT-trained Base Model

A key question in reward-guided generation is whether the improvements obtained by RL actually arise from policy optimization, or whether they can already be achieved by simply sampling candidates from the base SFT model and selecting the highest-reward TCR sequence from the candidate pool. In other words, if high-scoring sequences already exist within the original SFT distribution, then RL may provide little additional benefit beyond post-hoc reranking.

To investigate this, we performed a best-of-k experiment. For each epitope, we sampled ($k = 1, 2, 4, 8, 16, 32, 64$) candidate TCRs from the base SFT model and selected the highest-scoring sequence according to each reward function. We repeated 20 times to reduce sampling variance. This provides a non-RL baseline that isolates the effect of reward-based selection without modifying the underlying generative policy. The best-of-k experiment results are shown in Fig. A16. Comparison to RL-trained models are shown in Fig. A17, where metrics were computed per epitope, and the box plots summarize their distributions across epitopes, comparing post-hoc selection with RL-induced policy changes. For simplicity, we used best-of-16 selection results in this comparison as metrics tend to increase up to $k = 16$, then plateau, suggesting that $k = 16$ is the overall elbow point.

We observe that that the non-contrastive rewards outperform the base reward during SFT sampling, demonstrating their effectiveness. While the contrastive rewards do not show a clear trend, this is due to their bias towards selective TCRs. This demonstrates that our defended rewards are agnostic to sampling method. Our base method using RL demonstrates far more extreme reward hacking behavior, seen in the rapid decrease in evaluation scores when using RL with the base reward, despite using early stopping. We also observe that RL consistently increases the TCRMatch mean compared to post-hoc best-of-k sampling while reducing TCRMatch variance, showing the effect of the policy updates from RL.

A2 Supplementary Figures

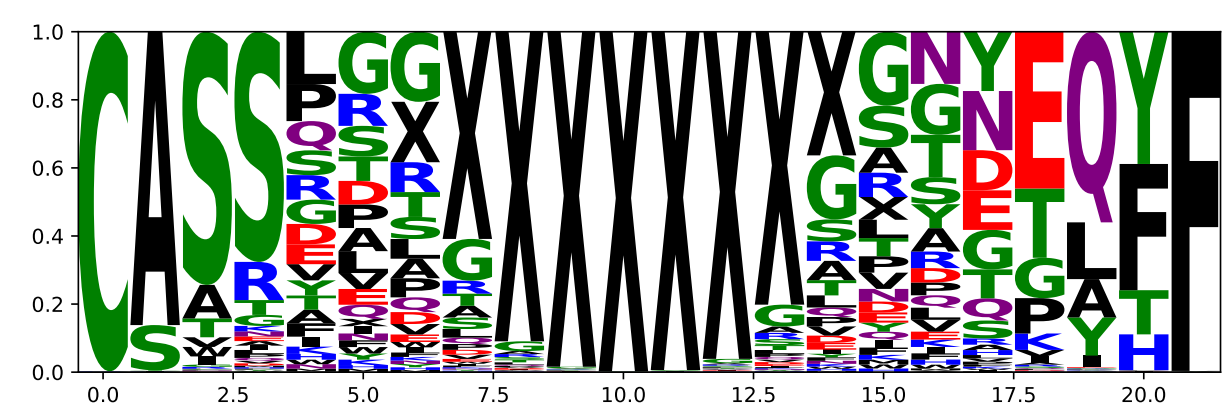

Figure A1: We include a Seqlogo of healthy TCRs for reference. Note the CASS and EQYF motifs.

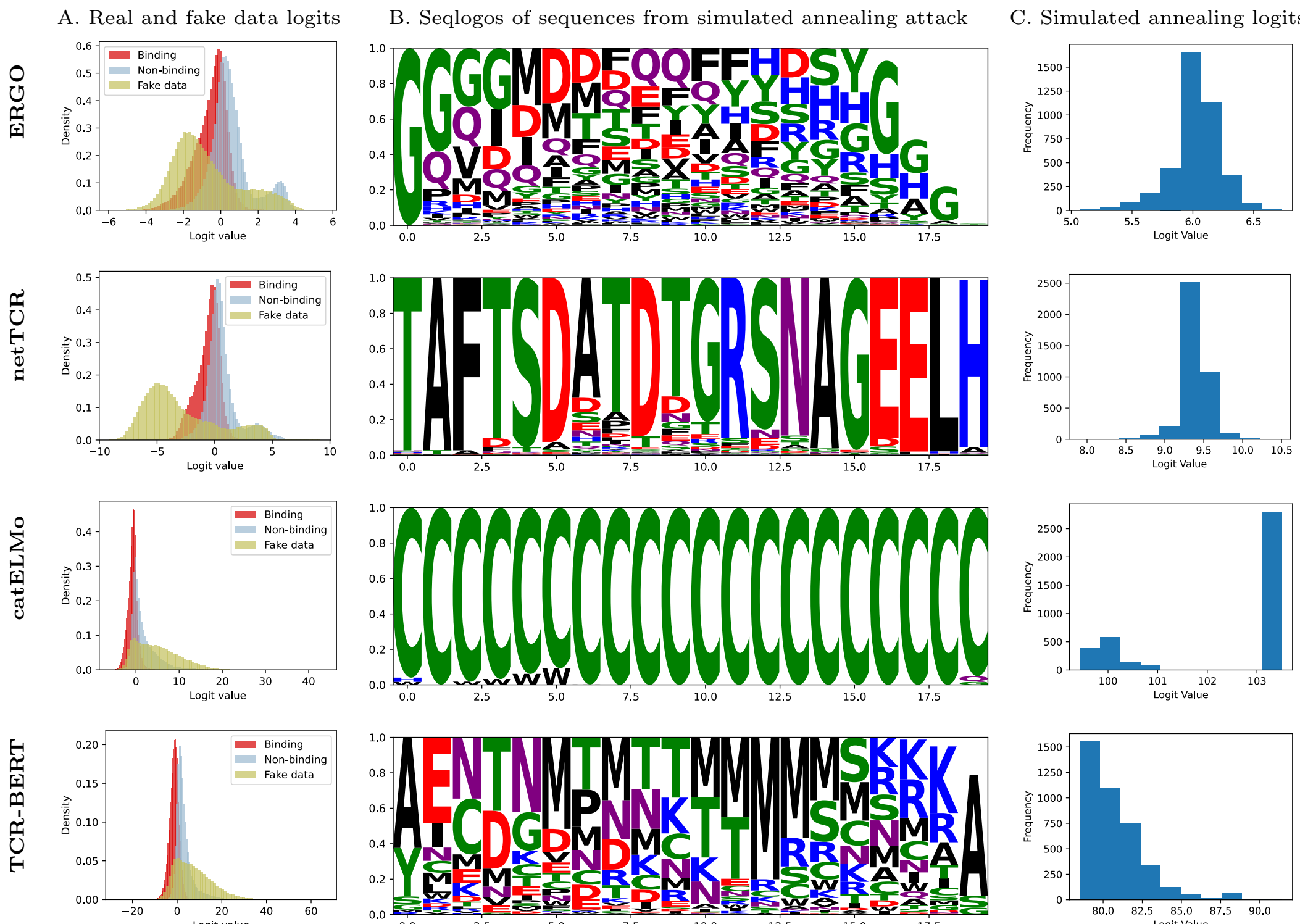

Figure A2: Evaluation of Reward Model Vulnerability. (A) Plots of logit distributions of real and fake data, (B) Seqlogos of sequences from the simulated annealing attack, and (C) logit distrbutions from the simulated annealing (SA) attack for different prediction models. A positive shift in the logit distribution is observed in TCR-BERT and catELMo, which are built on learned embeddings. Simulated annealing results show that both models are attackable, with each model outputting uncharacteristically high logit values for visibly abnormal sequences.

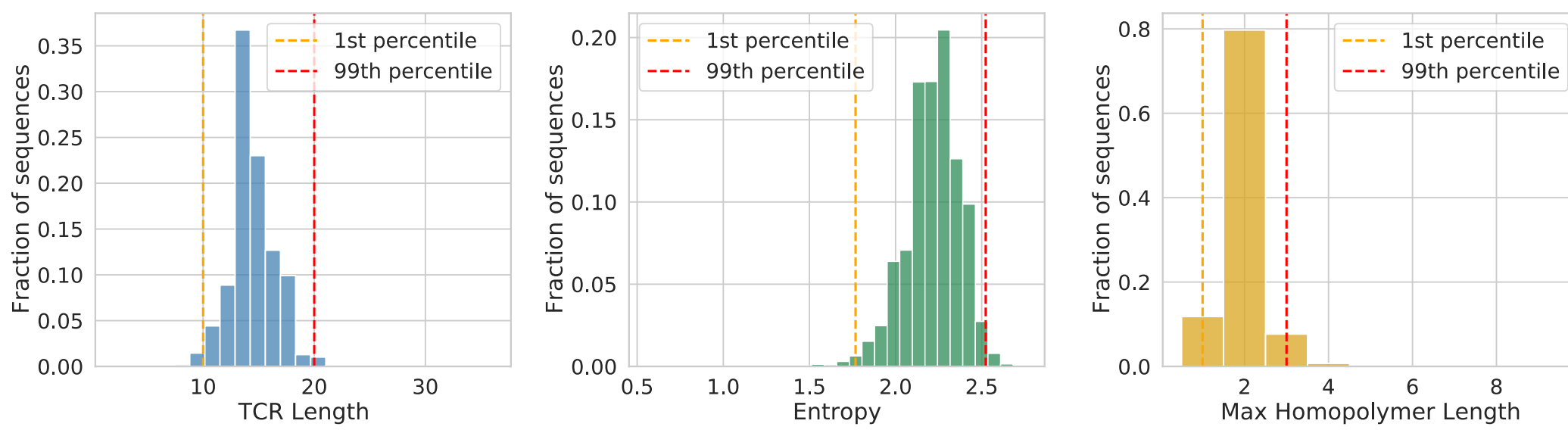

Figure A3: Distributions of known binder TCR statistics. Histograms of CDR3β length (left), amino-acid entropy (center), and maximum homopolymer length (right) computed from the training set of known binder TCRs. Vertical dashed lines indicate the 1st and 99th percentiles used to define the empirical biological thresholds applied during heuristic reward clipping.

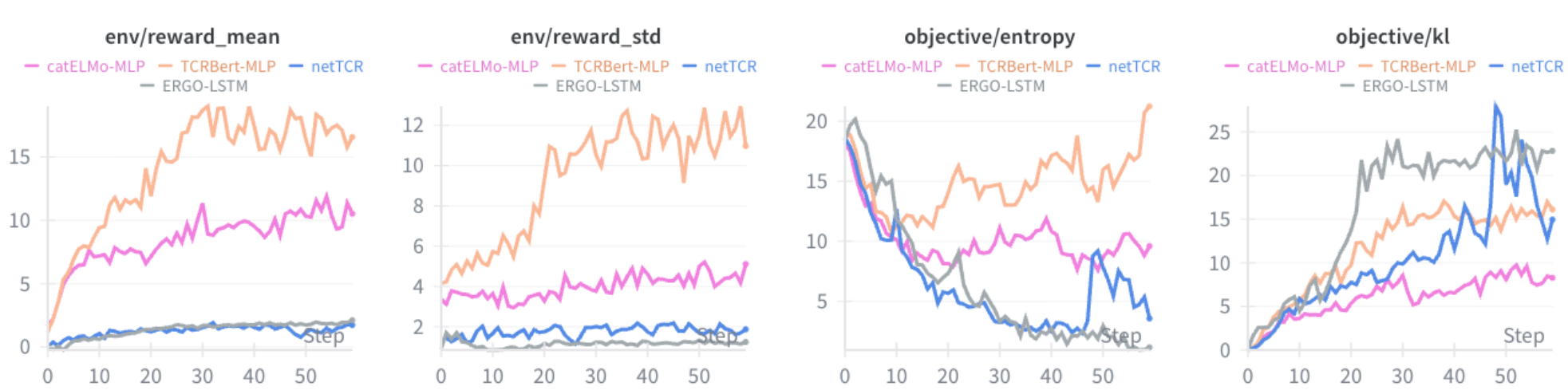

Figure A4: Training dynamics reveal reward hacking in baseline rewards. Mean reward, reward variance, policy entropy, and KL divergence across training steps for different base reward models (catELMo–MLP, TCR-BERT–MLP, netTCR, and ERGO–LSTM). Non–BLOSUM models (catELMo–MLP and TCRBERT–MLP) show rapid reward inflation, rising KL divergence, and collapsing entropy, characteristic of over-optimization of surrogate rewards. In contrast, BLOSUM-based models (netTCR and ERGO–LSTM) exhibit bounded rewards and smoother dynamics, indicating reduced susceptibility to reward hacking.

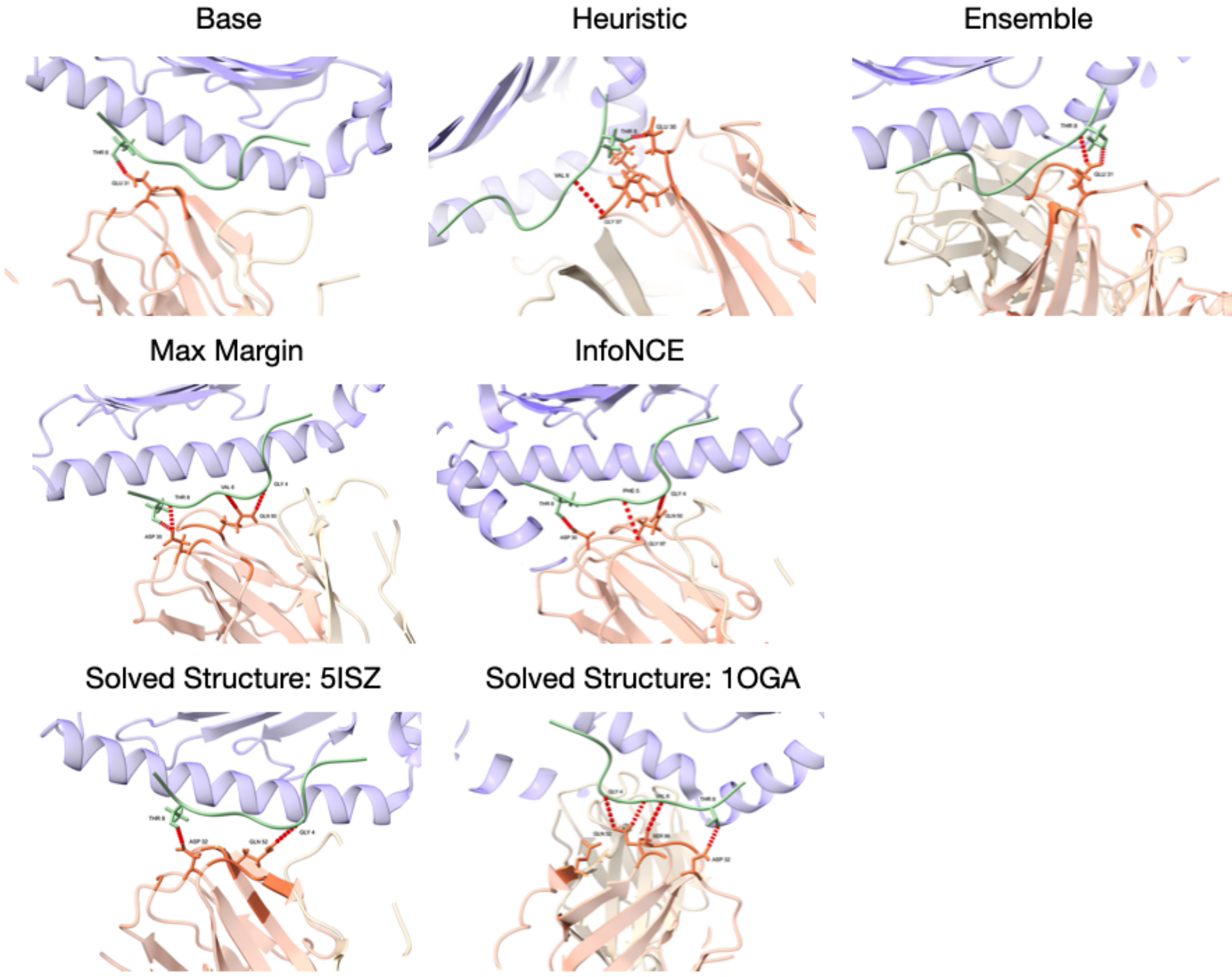

Figure A5: Comparative structural analysis of generated TCR–peptide interfaces across reward formulations. Representative complex models from the final training step (step 60) are shown for each reward design, alongside the experimentally solved GILGFVFTL-specific TCR–pMHC structures (PDB: 5ISZ and 1OGA) for reference. The peptide (green) and key interacting CDR3β residues (orange) are highlighted, with hydrogen bonds shown as red dashed lines. The Base model exhibits weak and poorly organized contacts relative to the solved structures, whereas the Heuristic and Ensemble models recover more cohesive interface geometries. The InfoNCE and Max-Margin formulations produce interfaces that closely resemble the native binding orientation, maintaining stable hydrogen-bond networks and peptide anchoring similar to experimentally determined complexes.

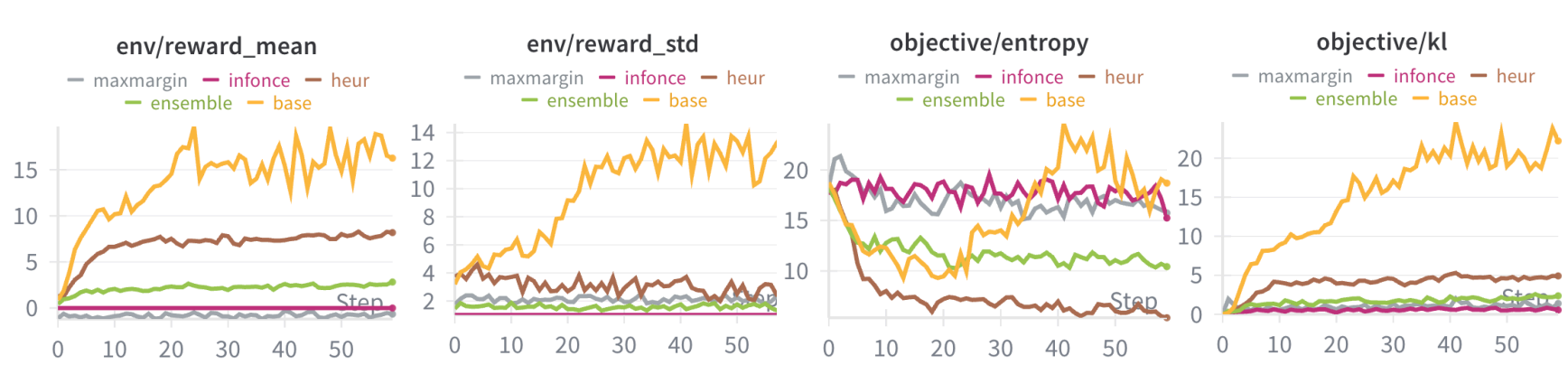

Figure A6: Training dynamics under a ProtGPT-initialized generator. We compare baseline and plug-and-play reward configurations across mean reward, reward variance, policy entropy, and KL divergence. Similar to the RITA backbone, the baseline exhibits reward inflation, entropy collapse, and KL escalation, whereas our plug-in reward designs stabilize optimization and prevent reward hacking.

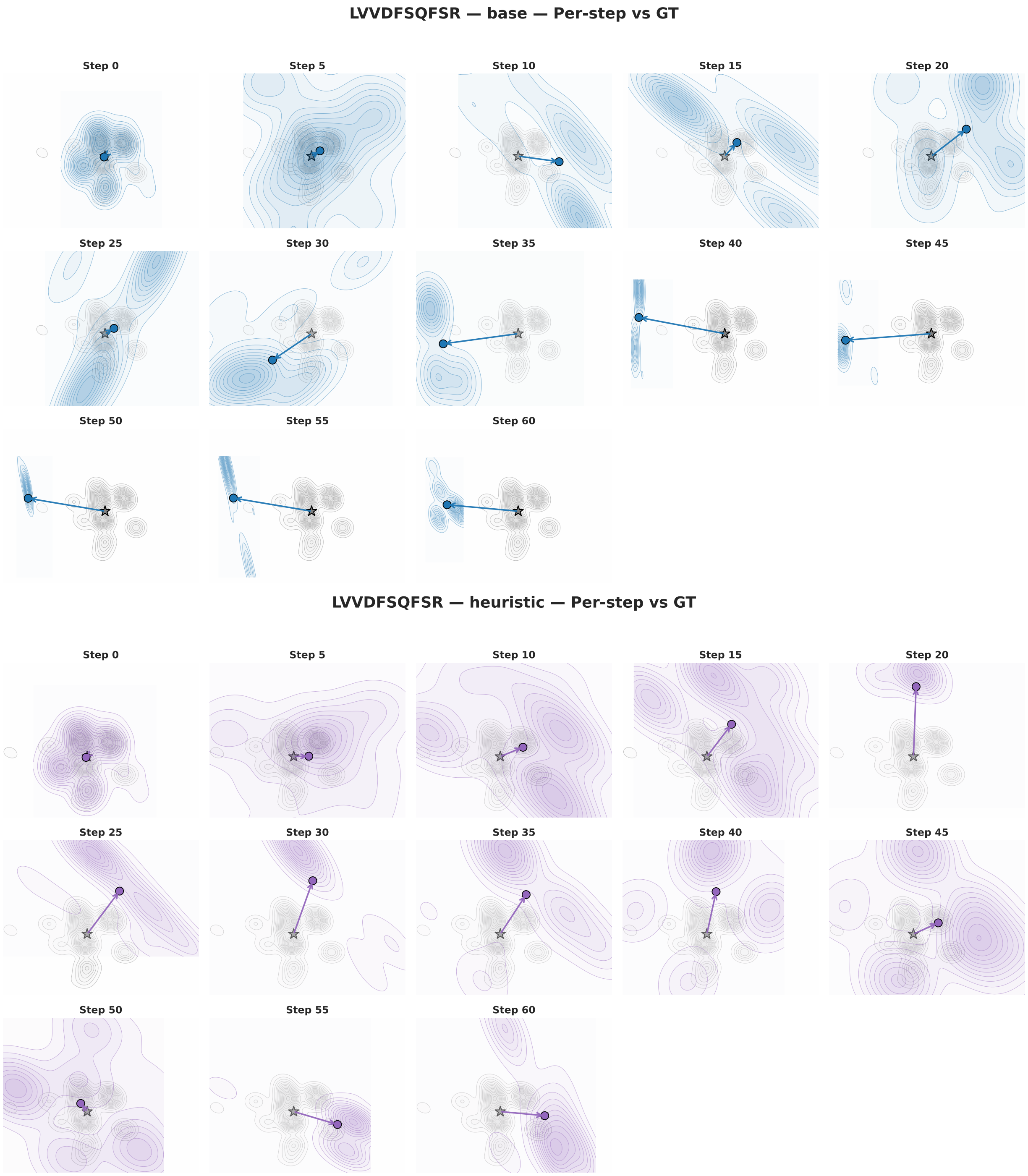

Figure A7: Per-step trajectory comparison for LVVDFSQFSR

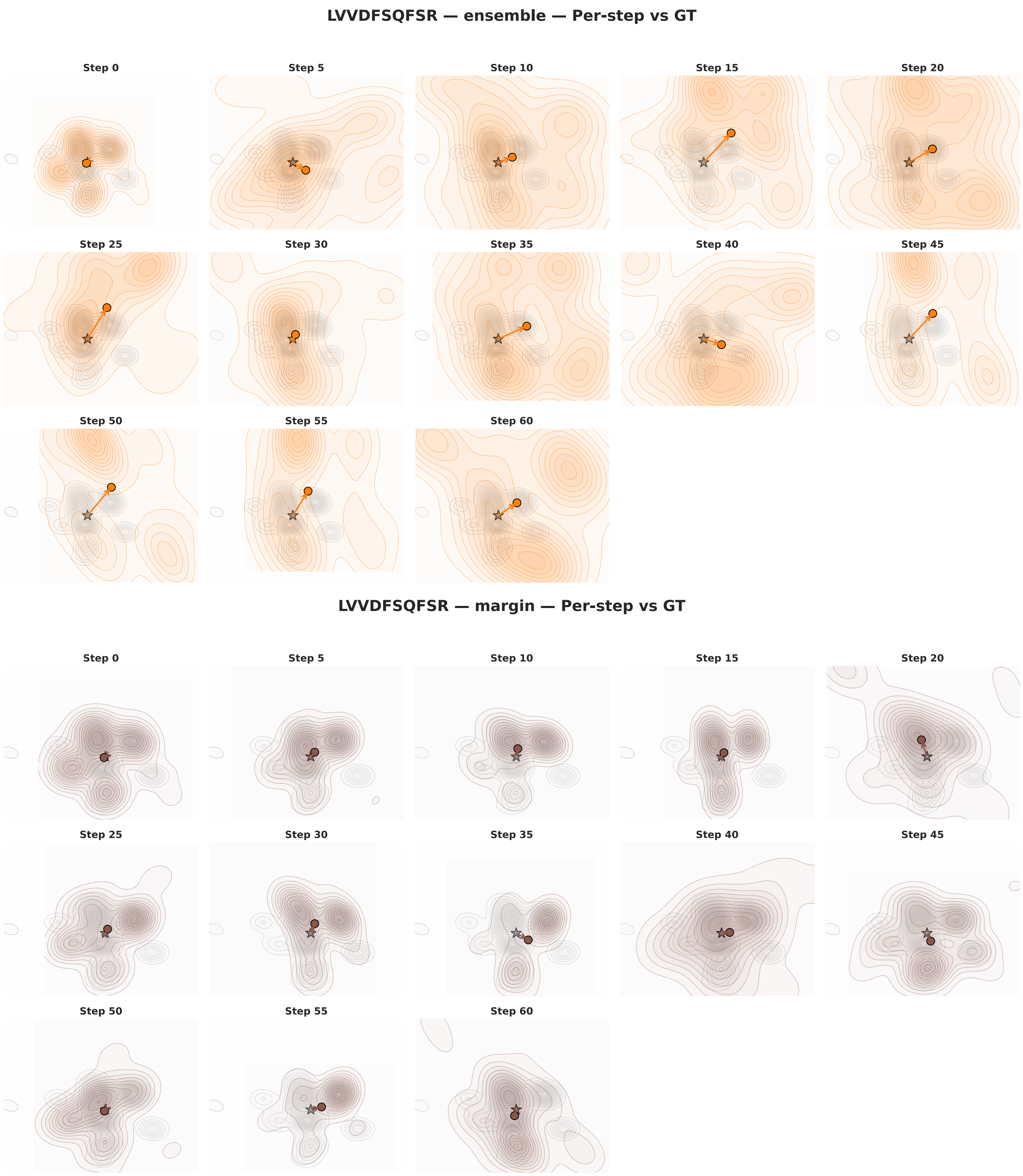

Figure A8: Per-step trajectory comparison for LVVDFSQFSR

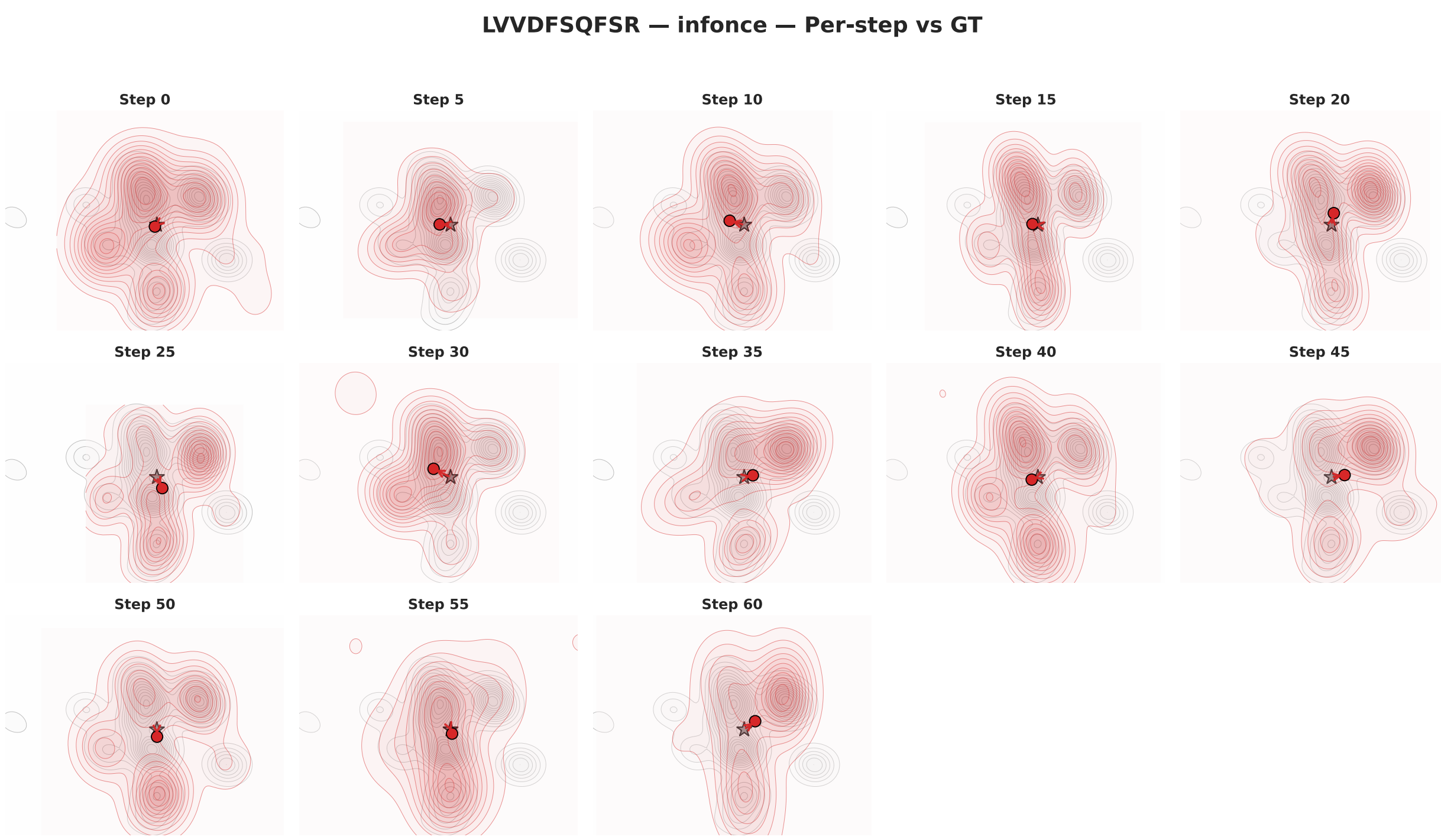

Figure A9: Per-step trajectory comparison for LVVDFSQFSR

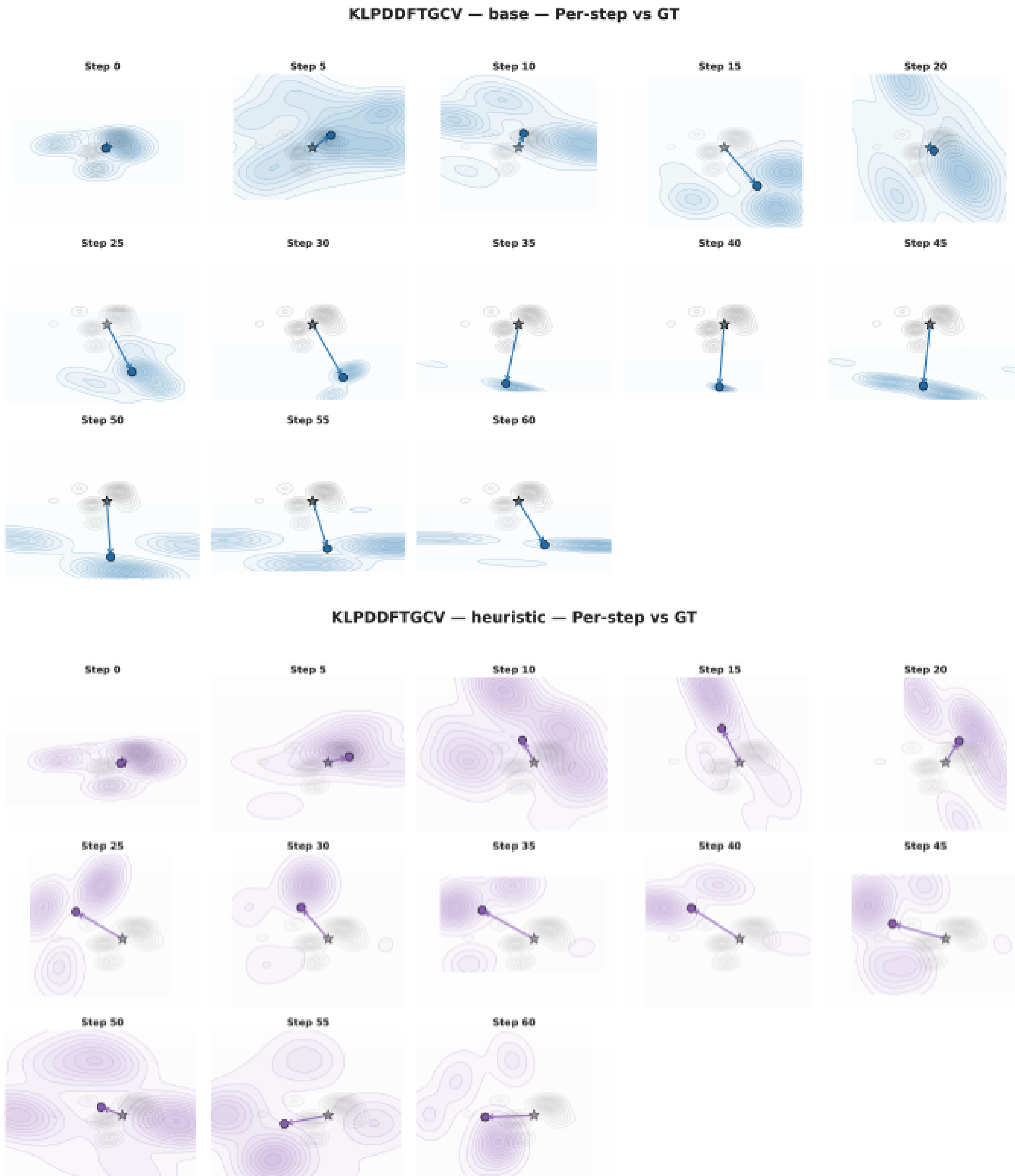

Figure A10: Per-step trajectory comparison for KLPDDFTGCV

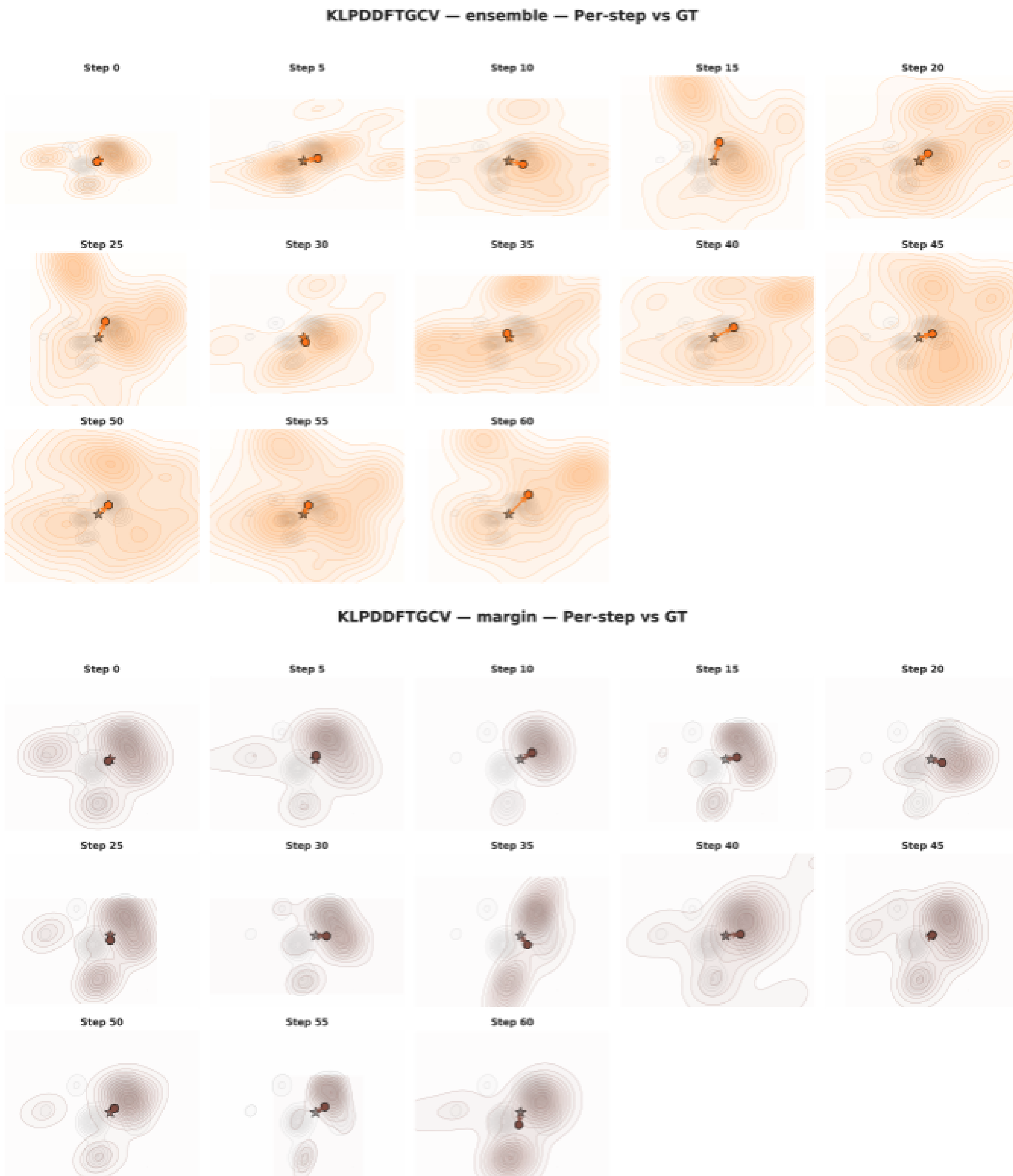

Figure A11: Per-step trajectory comparison for KLPDDFTGCV

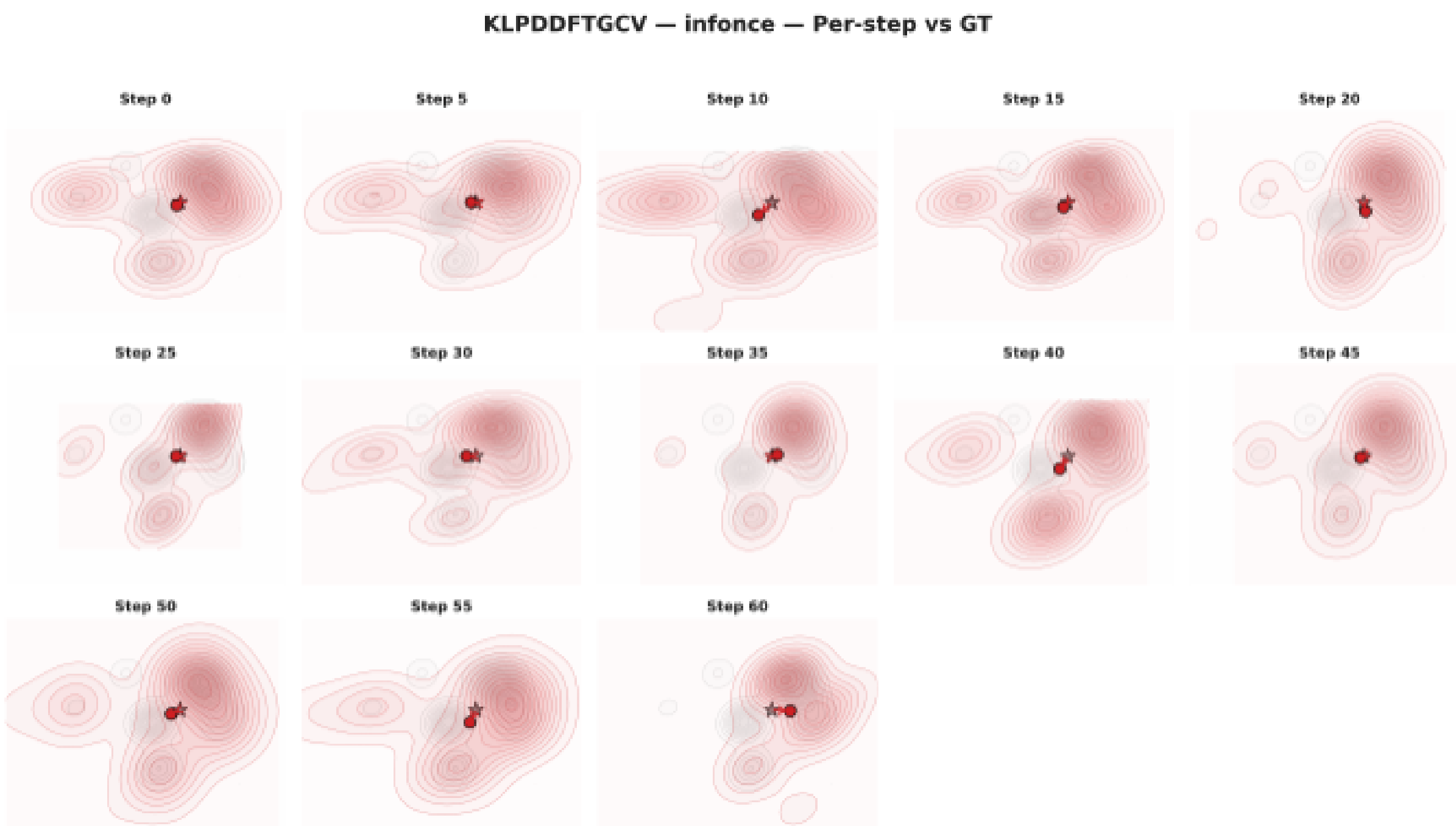

Figure A12: Per-step trajectory comparison for KLPDDFTGCV

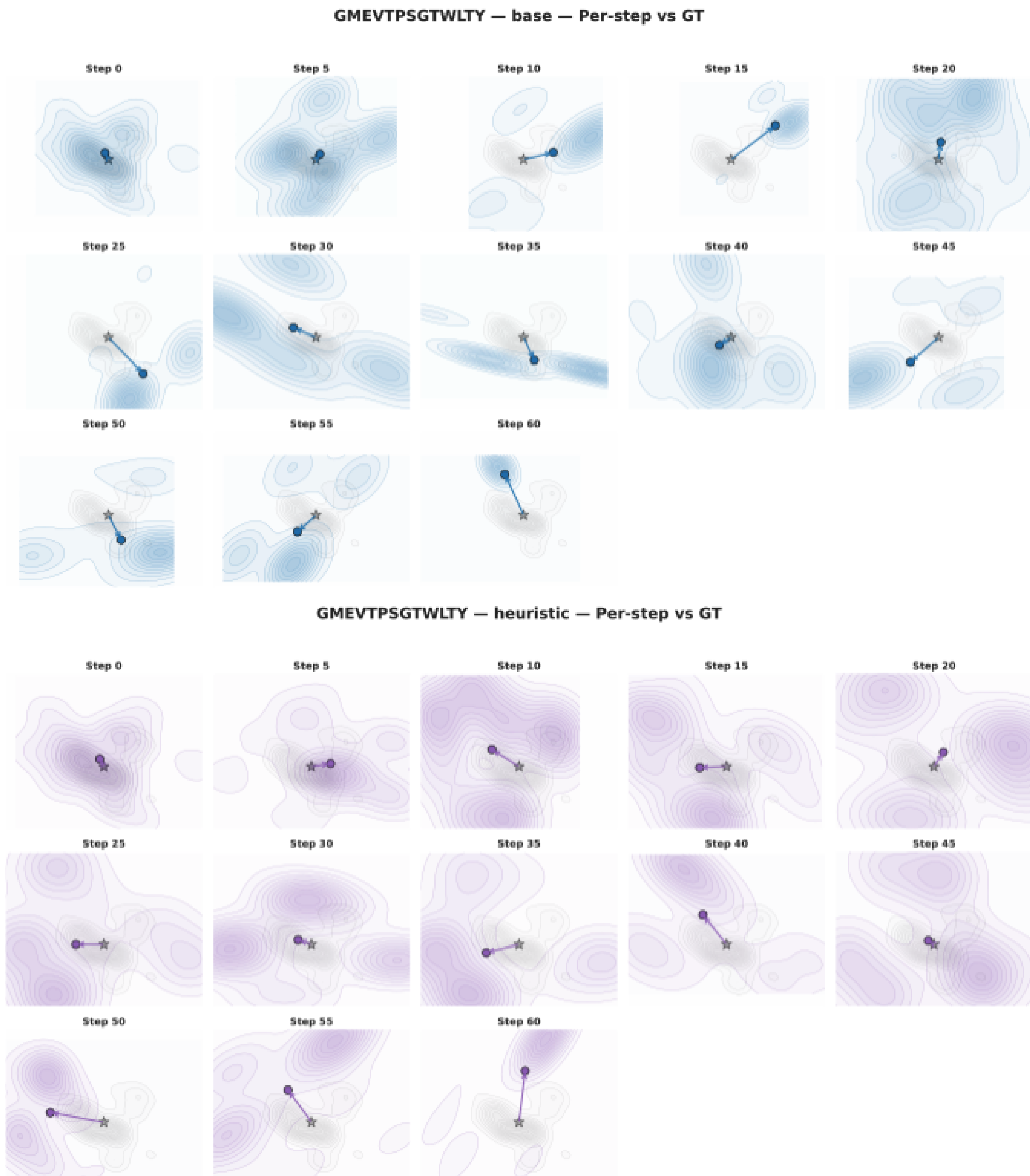

Figure A13: Per-step trajectory comparison for GMEVTPSGTWLTY

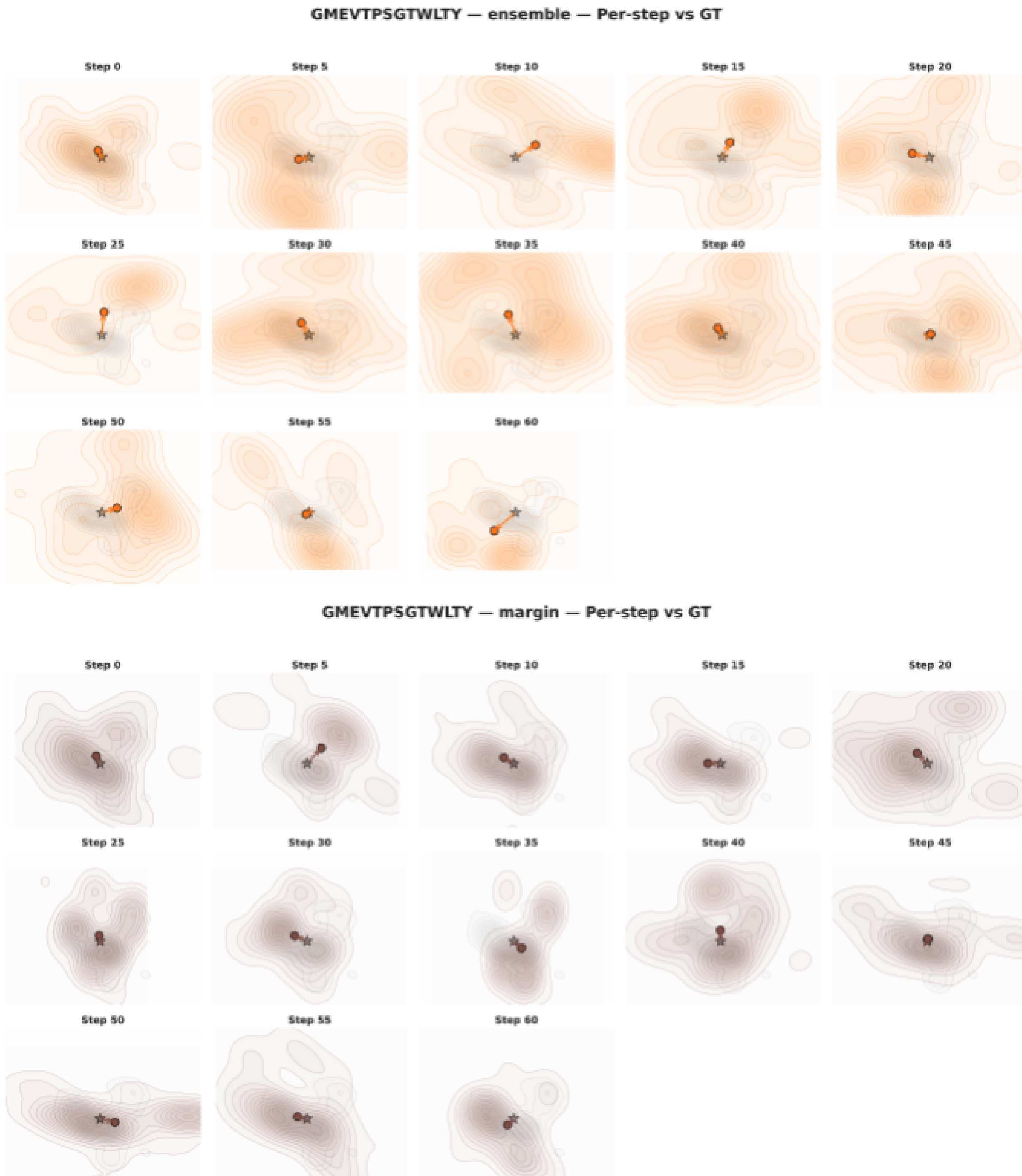

Figure A14: Per-step trajectory comparison for GMEVTPSGTWLTY

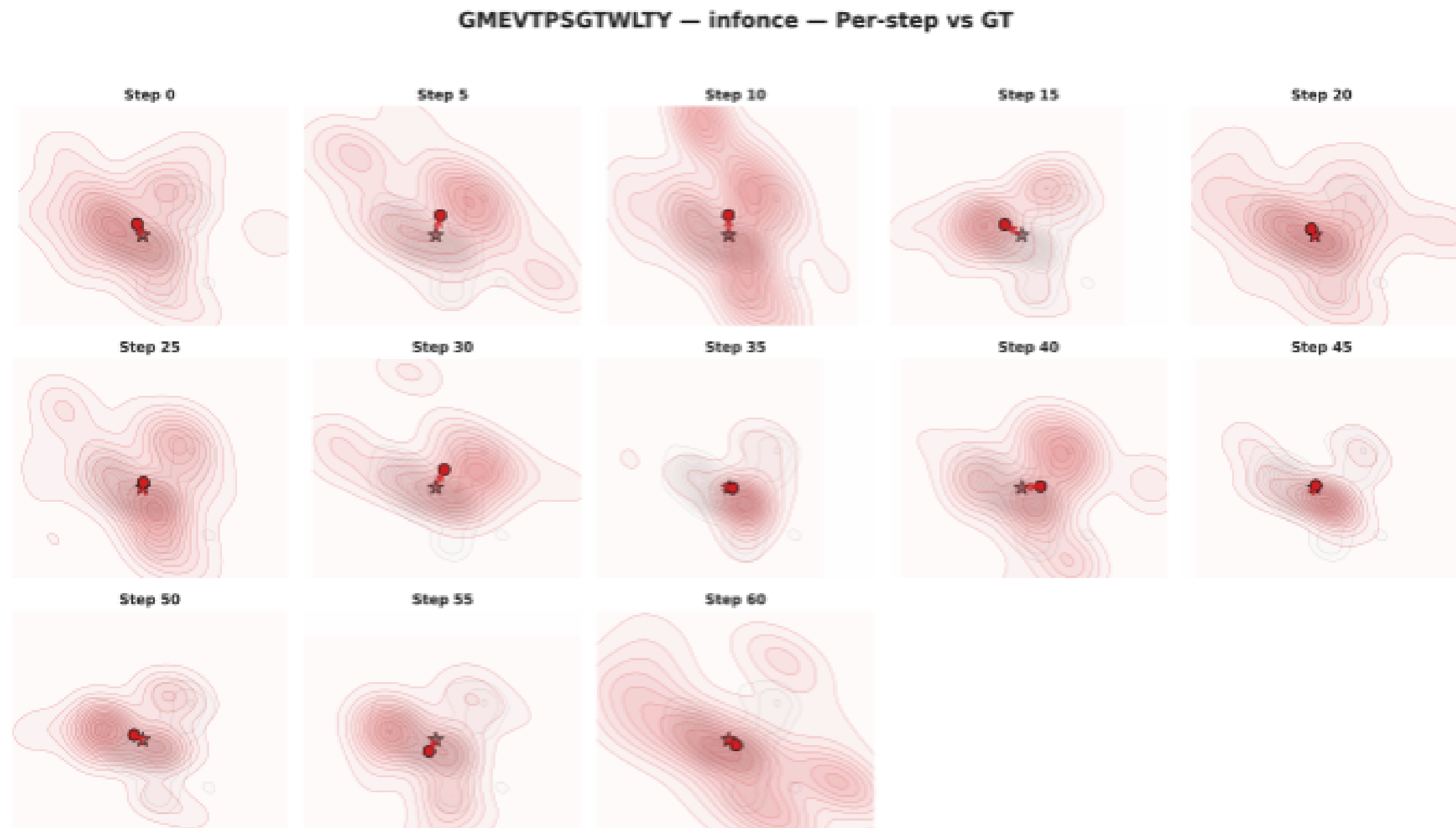

Figure A15: Per-step trajectory comparison for GMEVTPSGTWLTY

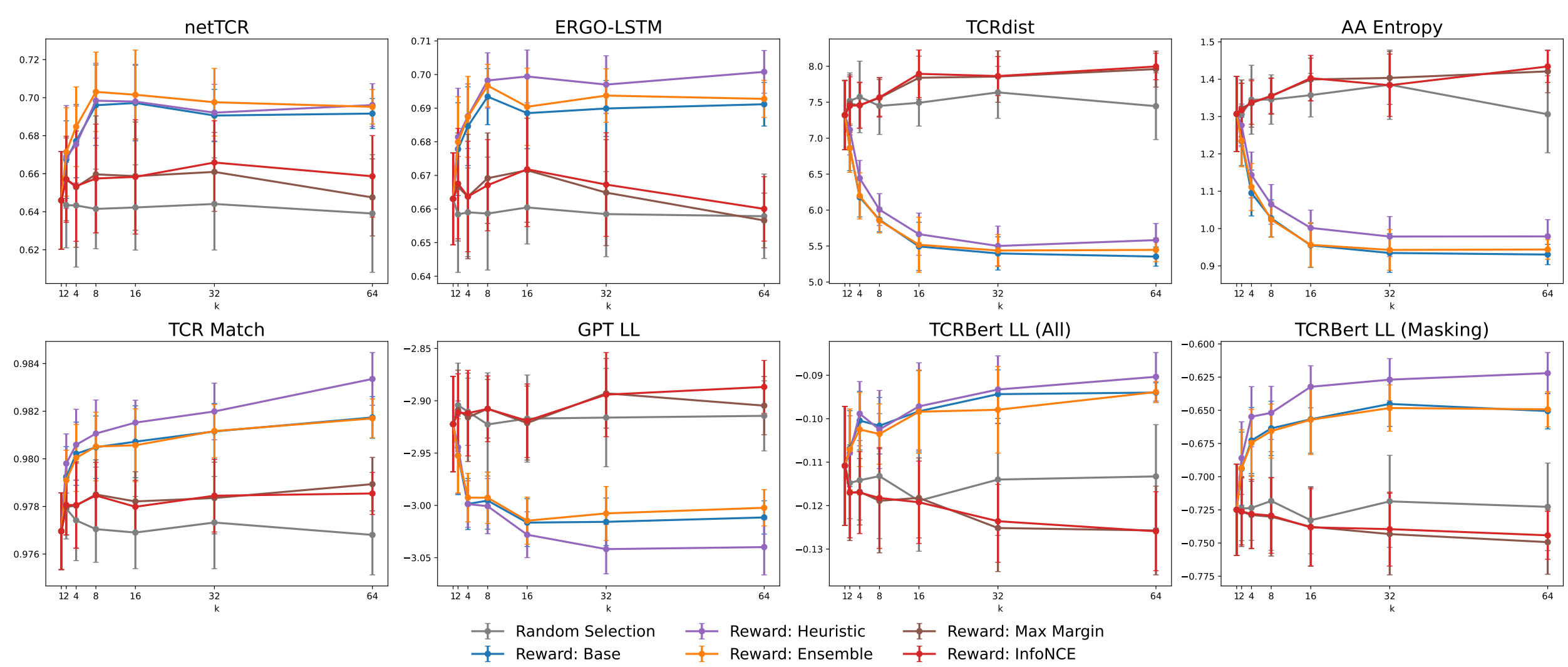

Figure A16: Best-of-k sampling results using different reward functions for post-hoc selection from the base SFT model distribution. For each epitope, k candidate TCRs were randomly sampled from the base model, and one sequence was selected either randomly or according to the corresponding reward function. Curves show the mean performance across repeated sampling runs, while error bars denote standard deviation across repeats.

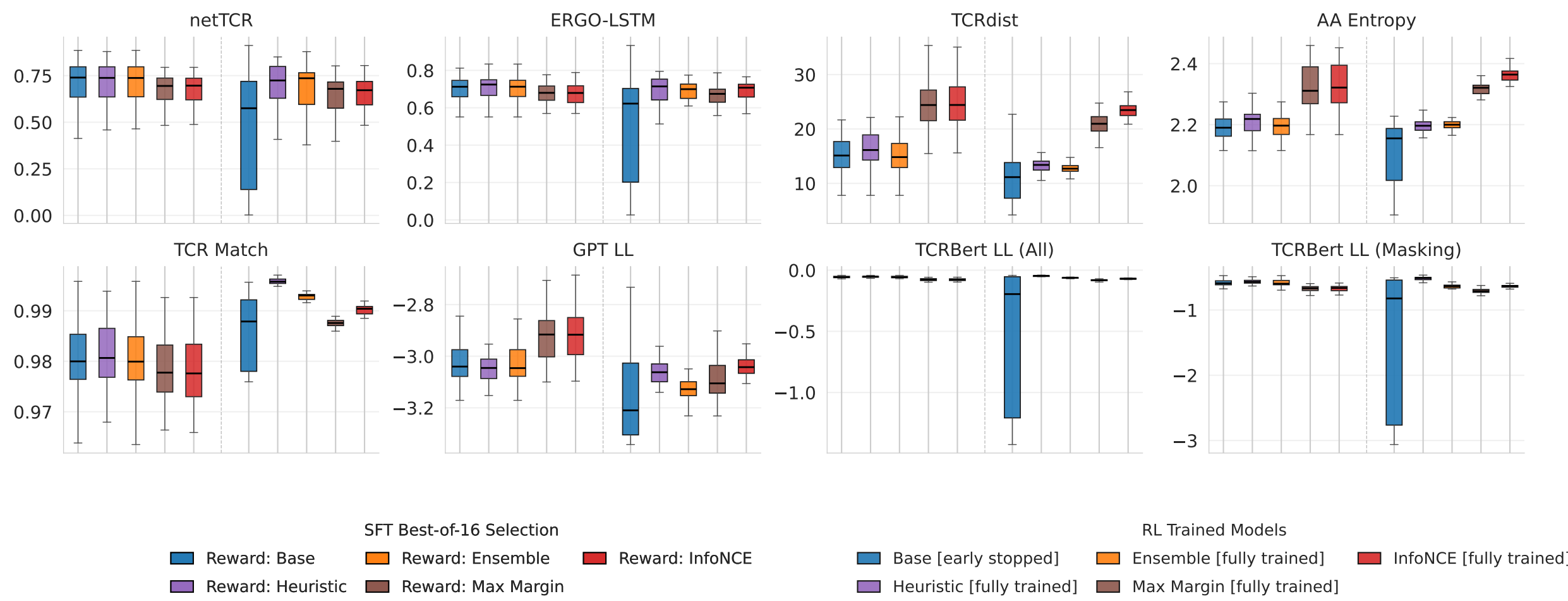

Figure A17: Comparison between Best-of-16 selection on the base SFT model and RL-trained models under per-epitope evaluation. The left boxplots show the distributions obtained by performing Best-of-16 sampling from the base SFT model and selecting sequences using different reward functions (Base, Heuristic, Ensemble, Max Margin, and InfoNCE). The right box plots show the corresponding distributions for fully trained RL models. For each epitope, evaluation metrics were first averaged across the generated TCR set. Box plots summarize the distributions across epitopes. Each color represents a different reward function used for output selection or RL traning.

Table A1: Paired Wilcoxon signed-rank tests comparing fully trained models against the early-stopped base model on per-epitope averaged metrics. For each epitope, metric values were first averaged across all generated TCRs, producing one paired observation per epitope for each model comparison. One-sided paired Wilcoxon signed-rank tests were then performed across epitopes to evaluate whether the fully trained models achieved significantly higher metric values than the base model. Reported p-values indicate the statistical significance of these improvements. * indicates significance at $p < 0.05$.

**Model**	**TCRMatch**	**ERGO-LSTM**	**netTCR**	**GPT LL**	**TCRBert LL-All**	**TCRBert LL-Masking**	**TCRdist**	**Entropy**
Ensemble	1.6$e-5$*	1.5$e-3$*	3.2$e-4$*	4.5$e-2$*	1.7$e-3$*	1.0$e-2$*	5.7$e-2$	9.3$e-8$*
Heuristic	3.7$e-9$*	4.4$e-4$*	6.8$e-5$*	4.0$e-4$*	2.4$e-6$*	1.1$e-8$*	5.2$e-2$	1.5$e-4$*
InfoNCE	2.0$e-2$*	1.5$e-3$*	8.4$e-3$*	1.5$e-4$*	3.8$e-3$*	7.3$e-3$*	1.1$e-8$*	3.7$e-9$*
Max Margin	2.2$e-1$	1.5$e-2$*	3.5$e-3$*	7.8$e-3$*	4.8$e-3$*	1.7$e-2$*	7.1$e-8$*	3.7$e-9$*

References

[1] Josh Abramson, Jonas Adler, Jack Dunger, Richard Evans, Tim Green, Alexander Pritzel, Olaf Ronneberger, Lindsay Willmore, Andrew J. Ballard, Joshua Bambrick, Sebastian W. Bodenstein, David A. Evans, Chia-Chun Hung, Michael O'Neill, David Reiman, Kathryn Tunyasuvunakool, Zachary Wu, Akvilė Žemgulytė, Eirini Arvaniti, Charles Beattie, Ottavia Bertolli, Alex Bridgland, Alexey Cherepanov, Miles Congreve, Alexander I. Cowen-Rivers, Andrew Cowie, Michael Figurnov, Fabian B. Fuchs, Hannah Gladman, Rishub Jain, Yousuf A. Khan, Caroline M. R. Low, Kuba Perlin, Anna Potapenko, Pascal Savy, Sukhdeep Singh, Adrian Stecula, Ashok Thillaisundaram, Catherine Tong, Sergei Yakneen, Ellen D. Zhong, Michal Zielinski, Augustin Žídek, Victor Bapst, Pushmeet Kohli, Max Jaderberg, Demis Hassabis, and John M. Jumper. Accurate structure prediction of biomolecular interactions with alphafold 3. *Nature*, 2024.

[2] Hanqun Cao, Hongrui Zhang, Junde Xu, Zhou Zhang, Lingdong Shen, Minghao Sun, Ge Liu, Jinbo Xu, Wu-Jun Li, Jinren Ni, Cesar de la Fuente-Nunez, Tianfan Fu, Yejin Choi, Pheng-Ann Heng, and Fang Wu. From supervision to exploration: What does protein language model learn during reinforcement learning? *arXiv preprint arXiv:2510.01571*, 2025.

[3] William D Chronister, Austin Crinklaw, Swapnil Mahajan, Randi Vita, Zeynep Koşaloğlu-Yalçın, Zhen Yan, Jason A Greenbaum, Leon E Jessen, Morten Nielsen, Scott Christley, et al. TCRMatch: predicting T-cell receptor specificity based on sequence similarity to previously characterized receptors. *Frontiers in Immunology*, 12:640725, 2021.

[4] Pradyot Dash, Andrew J Fiore-Gartland, Tomer Hertz, George C Wang, Shalini Sharma, Aisha Souquette, Jeremy Chase Crawford, E Bridie Clemens, Thi HO Nguyen, Katherine Kedzierska, et al. Quantifiable predictive features define epitope-specific T cell receptor repertoires. *Nature*, 547(7661):89–93, 2017.

[5] Carson Denison, Monte MacDiarmid, Fazl Barez, David Duvenaud, Shauna Kravec, Samuel Marks, Nicholas Schiefer, Ryan Soklaski, Alex Tamkin, Jared Kaplan, Buck Shlegeris, Samuel R. Bowman, Ethan Perez, and Evan Hubinger. Sycophancy to subterfuge: Investigating reward-tampering in large language models. *arXiv preprint arXiv:2406.10162*, 2024.

[6] Jacob Devlin, Ming-Wei Chang, Kenton Lee, and Kristina Toutanova. BERT: Pre-training of deep bidirectional transformers for language understanding. In Jill Burstein, Christy Doran, and Thamar Solorio, editors, *Proceedings of the 2019 Conference of the North American Chapter of the Association for Computational Linguistics: Human Language Technologies, Volume 1 (Long and Short Papers)*, pages 4171–4186, Minneapolis, Minnesota, June 2019. Association for Computational Linguistics.

[7] Yicheng Gao, Yuli Gao, Yuxiao Fan, Chengyu Zhu, Zhiting Wei, Chi Zhou, Guohui Chuai, Qinchang Chen, He Zhang, and Qi Liu. Pan-peptide meta learning for T-cell receptor–antigen binding recognition. *Nature Machine Intelligence*, 5(3):236–249, 2023.

[8] Charles AE Goodhart. Problems of monetary management: the UK experience. In *Monetary theory and practice: The UK experience*, pages 91–121. Springer, 1984.

[9] Daniel Hesslow, Niccoló Zanichelli, Pascal Notin, Iacopo Poli, and Debora Marks. RITA: a study on scaling up generative protein sequence models. *arXiv preprint arXiv:2205.05789*, 2022.

[10] Dhuvarakesh Karthikeyan, Sarah N. Bennett, Amy G. Reynolds, Benjamin G. Vincent, and Alex Rubinsteyn. Conditional generation of real antigen-specific T cell receptor sequences. *Nature Machine Intelligence*, 7(9):1494–1509, Sep 2025.

[11] Jacek Karwowski, Oliver Hayman, Xingjian Bai, Klaus Kiendlhofer, Charlie Griffin, and Joar Skalse. Goodhart's law in reinforcement learning. In B. Kim, Y. Yue, S. Chaudhuri, K. Fragkiadaki, M. Khan, and Y. Sun, editors, *International Conference on Representation Learning*, volume 2024, pages 24546–24576, 2024.

[12] S. Kirkpatrick, C. D. Gelatt, and M. P. Vecchi. Optimization by simulated annealing. *Science*, 220(4598):671–680, 1983.

[13] Victoria Krakovna, Jonathan Uesato, Vladimir Mikulik, Matthew Rahtz, Tom Everitt, Ramana Kumar, Zac Kenton, Jan Leike, and Shane Legg. Specification gaming: the flip side of AI ingenuity, 2024.

[14] Julia Koehler Leman, Brian D. Weitzner, Steven M. Lewis, Jared Adolf-Bryfogle, Nawsad Alam, Rebecca F. Alford, Melanie Aprahamian, David Baker, Kyle A. Barlow, Patrick Barth, et al. Macromolecular modeling and design in rosetta: recent methods and frameworks. *Nature Methods*, 2020.

[15] David Manheim and Scott Garrabrant. Categorizing variants of Goodhart's law. *arXiv preprint arXiv:1803.04585*, 2018.

[16] Elaine C. Meng, Thomas D. Goddard, Eric F. Pettersen, Greg S. Couch, Zach J. Pearson, John H. Morris, and Thomas E. Ferrin. Ucsf chimerax: Tools for structure building and analysis. *Tools for Protein Science*, 2023.

[17] Alessandro Montemurro, Viktoria Schuster, Helle Rus Povlsen, Amalie Kai Bentzen, Vanessa Jurtz, William D Chronister, Austin Crinklaw, Sine R Hadrup, Ole Winther, Bjoern Peters, et al. NetTCR-2.0 enables accurate prediction of TCR-peptide binding by using paired TCRα and β sequence data. *Communications biology*, 4(1):1060, 2021.

[18] Se Yeon Seo and Je-Keun Rhee. Tcr-epidiff: solving dual challenges of tcr generation and binding prediction. *Bioinformatics*, 41(Supplement 1):125–132, 07 2025.

[19] Zachary Sethna, Yuval Elhanati, Curtis G Callan Jr, Aleksandra M Walczak, and Thierry Mora. OLGA: fast computation of generation probabilities of B-and T-cell receptor amino acid sequences and motifs. *Bioinformatics*, 35(17):2974–2981, 2019.

[20] Mikhail Shugay, Dmitriy V Bagaev, Ivan V Zvyagin, Renske M Vroomans, Jeremy Chase Crawford, Garry Dolton, Ekaterina A Komech, Anastasiya L Sycheva, Anna E Koneva, Evgeniy S Egorov, et al. VDJdb: a curated database of T-cell receptor sequences with known antigen specificity. *Nucleic Acids Research*, 46(D1):D419–D427, 2018.

[21] Ido Springer, Hanan Besser, Nili Tickotsky-Moskovitz, Shirit Dvorkin, and Yoram Louzoun. Prediction of specific TCR-peptide binding from large dictionaries of TCR-peptide pairs. *Frontiers in immunology*, 11:1803, 2020.

[22] Filippo Stocco, Maria Artigues-Lleixa, Andrea Hunklinger, Talal Widatalla, Marc Guell, and Noelia Ferruz. Guiding generative protein language models with reinforcement learning. *arXiv preprint arXiv:2412.12979*, 2025.

[23] Nili Tickotsky, Tal Sagiv, Jaime Prilusky, Eric Shifrut, and Nir Friedman. McPAS-TCR: a manually curated catalogue of pathology-associated T cell receptor sequences. *Bioinformatics*, 33(18):2924–2929, 2017.

[24] Randi Vita, Swapnil Mahajan, James A Overton, Sandeep Kumar Dhanda, Sheridan Martini, Jason R Cantrell, Daniel K Wheeler, Alessandro Sette, and Bjoern Peters. The immune epitope database (IEDB): 2018 update. *Nucleic Acids Research*, 47(D1):D339–D343, 2019.

[25] Talal Widatalla, Rafael Rafailov, and Brian Hie. Aligning protein generative models with experimental fitness via direct preference optimization. *bioRxiv*, 2024.

[26] Kevin Wu, Kathryn E. Yost, Bence Daniel, Julia A. Belk, Yu Xia, Takeshi Egawa, Ansuman Satpathy, Howard Y. Chang, and James Zou. TCR-BERT: learning the grammar of T-cell receptors for flexible antigen-binding analyses. *bioRxiv*, 2021.

[27] Junhao Xiong, Hunter Nisonoff, Maria Lukarska, Ishan Gaur, Luke M. Oltrogge, David F. Savage, and Jennifer Listgarten. Guide your favorite protein sequence generative model. *arXiv preprint arXiv:2505.04823*, 2025.

[28] Jiannan Yang, Bing He, Yu Zhao, Feng Jiang, Zhonghuang Wang, Yixin Guo, Zhimeng Xu, Bo Yuan, Jiangning Song, Qingpeng Zhang, and Jianhua Yao. De novo generation of T-cell receptors with desired epitope-binding property by leveraging a pre-trained large language model. *bioRxiv*, 2023.

[29] Pengfei Zhang, Seojin Bang, Michael Cai, and Heewook Lee. Context-aware amino acid embedding advances analysis of TCR-epitope interactions. *eLife*, April 2023.

[30] Pengfei Zhang, Seojin Bang, and Heewook Lee. Self-contemplating in-context learning enhances T cell receptor generation for novel epitopes. In *Machine Learning in Computational Biology*. PMLR, 2025.

[31] Pengfei Zhang, Hao Mei, Seojin Bang, and Heewook Lee. Iterative attack-and-defend framework for improving TCR-epitope binding prediction models. *Bioinformatics*, 41(Supplement_1):i429–i438, 2025.

[32] Zhenghong Zhou, Junwei Chen, Shenggeng Lin, Liang Hong, Dong-Qing Wei, and Yi Xiong. GRATCR: Epitope-specific T cell receptor sequence generation with data-efficient pre-trained models. *IEEE Journal of Biomedical and Health Informatics*, 29(3):2271–2283, 2025.
